# Metagenomic Investigation of Ticks From Kenyan Wildlife Reveals Diverse Microbial Pathogens and New Country Pathogen Records

**DOI:** 10.3389/fmicb.2022.932224

**Published:** 2022-07-01

**Authors:** Koray Ergunay, Mathew Mutinda, Brian Bourke, Silvia A. Justi, Laura Caicedo-Quiroga, Joseph Kamau, Samson Mutura, Irene Karagi Akunda, Elizabeth Cook, Francis Gakuya, Patrick Omondi, Suzan Murray, Dawn Zimmerman, Yvonne-Marie Linton

**Affiliations:** ^1^Walter Reed Biosystematics Unit (WRBU), Smithsonian Institution Museum Support Center, Suitland, MD, United States; ^2^One Health Branch, Walter Reed Army Institute of Research (WRAIR), Silver Spring, MD, United States; ^3^Department of Medical Microbiology, Virology Unit, Faculty of Medicine, Hacettepe University, Ankara, Turkey; ^4^Department of Entomology, Smithsonian Institution, National Museum of Natural History (NMNH), Washington, DC, United States; ^5^Kenya Wildlife Services Corporation, Nairobi, Kenya; ^6^One Health Centre, Institute of Primate Research (IPR), Nairobi, Kenya; ^7^International Livestock Research Institute (ILRI), Nairobi, Kenya; ^8^Wildlife Research and Training Institute (WRTI), Naivasha, Kenya; ^9^Global Health Program, Smithsonian Conservation Biology Unit, Fort Royal, VA, United States; ^10^Department of Epidemiology of Microbial Disease, Yale School of Public Health, New Haven, CT, United States

**Keywords:** tick, metagenomics, wildlife, pathogen, surveillance

## Abstract

Focusing on the utility of ticks as xenosurveillance sentinels to expose circulating pathogens in Kenyan drylands, host-feeding ticks collected from wild ungulates [buffaloes, elephants, giraffes, hartebeest, impala, rhinoceros (black and white), zebras (Grévy’s and plains)], carnivores (leopards, lions, spotted hyenas, wild dogs), as well as regular domestic and Boran cattle were screened for pathogens using metagenomics. A total of 75 host-feeding ticks [*Rhipicephalus* (97.3%) and *Amblyomma* (2.7%)] collected from 15 vertebrate taxa were sequenced in 46 pools. Fifty-six pathogenic bacterial species were detected in 35 pools analyzed for pathogens and relative abundances of major phyla. The most frequently observed species was *Escherichia coli* (62.8%), followed by *Proteus mirabilis* (48.5%) and *Coxiella burnetii* (45.7%). *Francisella tularemia* and Jingmen tick virus (JMTV) were detected in 14.2 and 13% of the pools, respectively, in ticks collected from wild animals and cattle. This is one of the first reports of JMTV in Kenya, and phylogenetic reconstruction revealed significant divergence from previously known isolates and related viruses. Eight fungal species with human pathogenicity were detected in 5 pools (10.8%). The vector-borne filarial pathogens (*Brugia malayi, Dirofilaria immitis, Loa loa*), protozoa (*Plasmodium* spp., *Trypanosoma cruzi*), and environmental and water-/food-borne pathogens (*Entamoeba histolytica, Encephalitozoon intestinalis, Naegleria fowleri, Schistosoma* spp., *Toxoplasma gondii*, and *Trichinella spiralis*) were detected. Documented viruses included human mastadenovirus C, Epstein-Barr virus and bovine herpesvirus 5, Trinbago virus, and Guarapuava tymovirus-like virus 1. Our findings confirmed that host-feeding ticks are an efficient sentinel for xenosurveillance and demonstrate clear potential for wildlife-livestock-human pathogen transfer in the Kenyan landscape.

## Introduction

Tick-borne infections account for a major portion of all vector-borne diseases in many developed and underdeveloped countries, with significant health, economic, and food security consequences (Rochlin and Toledo, 2020). The incidence of tick-borne infections is increasing due to environmental changes at both local and global scales, resulting in changing habitats and geographical repartition of ticks, with subsequent novel pathogen exposure in naive populations (Ogden et al., 2013).

Ticks can transmit a diverse array of microbial pathogens including viruses, bacteria, and protozoans to humans and susceptible animals (Rochlin and Toledo, 2020). Tick-borne infections in humans are of zoonotic origin, with pathogens maintained in natural cycles involving various domestic and wild animal hosts (Wikel, 2018). Timely identification of the circulating pathogens and assessment of potential public health threats rely on effective surveillance, especially considering that most zoonoses originate from wildlife (Woolhouse and Gowtage-Sequeria, 2005; Miller et al., 2013). Many tick-borne agents of concern might already be circulating in wildlife awaiting detection. Microbial pathogens may be overlooked until the emergence of a case cluster or a local epidemic. An effective surveillance strategy should be based on surveying a wide range of pathogens and hosts, prior to the spread and symptomatic disease. The availability of metagenomic sequencing platforms and approaches offers great advantages and possibilities to explore microbial diversity (Brinkmann et al., 2016). Metagenomic investigations in tick vectors collected from wildlife hosts provide opportunities to obtain spatial and temporal microbial genome data as well as from tick-borne pathogens, and are likely to facilitate predictions on possible emergents.

The Republic of Kenya is a large sub-Saharan country in eastern Africa, with an area of 580,367 square kilometers and a population of over 47 million (Kenya National Bureau of Statistics, 2021). In Kenya, the pastoralist population make up around 20% of the population with an agricultural land use of nearly 50% (State of Wildlife Conservancies in Kenya Report, 2016). National parks, nature reserves, and conservancies account for about 11% of the Kenyan landmass with increased human and livestock populations in peripheries (Wellde et al., 1989: Ogutu et al., 2016). These trends facilitate interactions between livestock, humans, and wildlife in a complex environment, increasing the opportunity for pathogen spill-over events (Okal et al., 2020). Several tick-borne bacterial, viral, and parasitic agents have been documented in Kenya, including agents of anaplasmosis, ehrlichiosis, rickettsiosis, Crimean-Congo hemorrhagic fever (CCHF), babesiosis, and theileriosis (Omondi et al., 2017; Morrison et al., 2020; Okal et al., 2020; Chiuya et al., 2021; Getange et al., 2021; Peter et al., 2021), with occasional reporting of novel pathogens (Mwamuye et al., 2017). However, reports from wildlife are relatively rare and often focus on well-documented bacterial agents. This study aims to screen ticks collected from wildlife hosts using an unbiased metagenomic approach, to investigate a broad spectrum of circulating microbial pathogens in Kenya dryland ecosystems.

## Materials and Methods

### Tick Specimens

**FIGURE 1:**
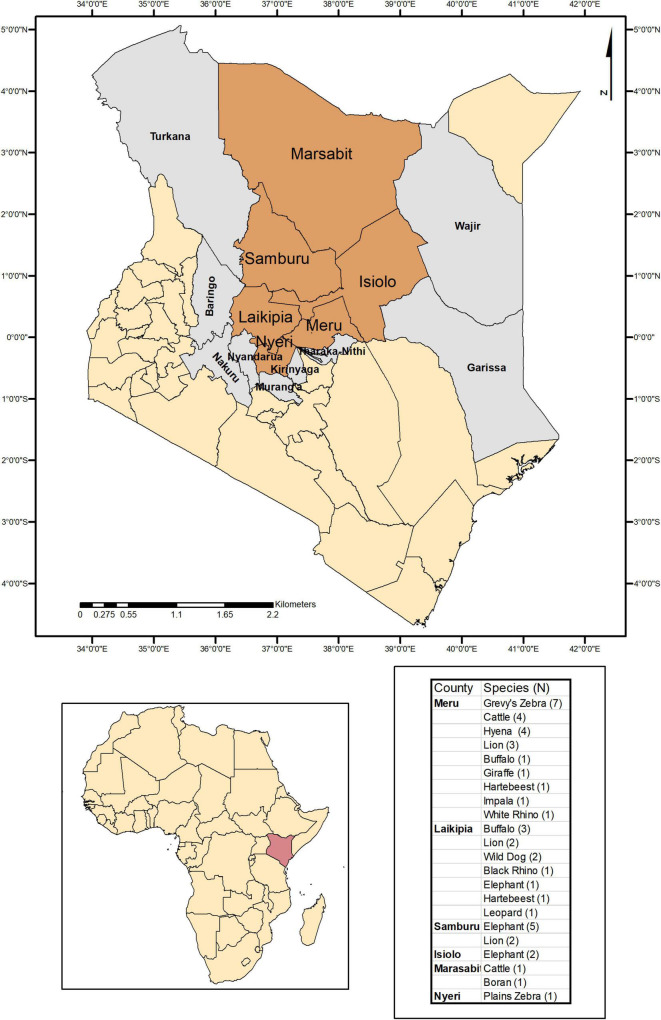
Map indicating the counties where ticks were collected and sampled when feeding on domestic and wild animals in Kenya.

Tick specimens were collected by the Kenya Wildlife Service (KWS) from animals in wildlife conservancy sites in Isiolo county, Kalama, Lewa, Ol Pejeta, Samburu, Sangare gardens, Sarara, Solio ranch and Oljogi, between October 2013 and April 2019 (Figure 1). The host animals included black rhinoceros (*Diceros bicornis*), buffalo (*Syncerus caffer*), elephant (*Loxodonta africana*), giraffe (*Giraffa camelopardalis*), Grévy’s zebra (*Equus grevyi*), hartebeest (*Alcelaphus buselaphus*), impala (*Aepyceros melampus*), leopard (*Panthera pardus*), lion (*Panthera leo*), plains zebra (*Equus quagga*), spotted hyena (*Crocuta crocuta*), white rhinoceros (*Ceratotherium simum*), and wild dog (*Lycaon pictus*), as well as farmed Boran (*Bos indicus*) and cattle (*Bos taurus*) (Table 1). The Boran and cattle were kept in ranches without fencing, in direct contact with wildlife. The ticks were collected opportunistically from wild animals immobilized by the KWS for veterinary intervention, and ticks were kept in 70% ethanol and stored at −80*^o^*C prior to processing. Information on the collection site, date, and the host was recorded. Ticks were morphologically identified as *Rhipicephalus* spp. or *Amblyomma* spp. and pooled according to individual host and genera, prior to pooling and DNA extraction (Table 1).

**TABLE 1:** Overview of the tick specimens and their hosts included in the study.

Host	Pool Code	Filtered Read Count	Pooled Individuals	Identification	Ticks from Host
Elephant (n:7, 15.2%)	#71	8,122	1♀	*Rhipicephalus* spp.	8/75 (10.6%)
	#65	6,426	1♂		
	#52	564,490	2♂		
	#48	71,616	1♀		
	#42	4,882	1♂		
	#41	3,294	1♂		
	#6	31,338	1♂		
Lion (n:7, 15.2%)	#53	1,631,344	1♀	*Rhipicephalus* spp.	7/75 (9.3%)
	#47	2,851,375	1♀		
	#45	1,350	1♀		
	#43	216,970	1♀		
	#23	15,276	1♂		
	#22	918	1♂	*Amblyomma* spp.	
	#10	84,976	1♀	*Rhipicephalus* spp.	
Grévy’s zebra (n:7, 15.2%)	#83	144,564	1♂	*Rhipicephalus* spp.	7/75 (9.3%)
	#78	3,552	1♀		
	#70	441,284	1♂		
	#69	28,338	1♂		
	#67	1,544	1♂		
	#25	5,296	1♀		
	#13	2,548	1♂		
Buffalo (n:4, 8.7%)	#84	43,042	5 (3♀2♂)	*Rhipicephalus* spp.	21/75 (28%)
	#58	33,668	1♂		
	#9	34,960	7 (4♀3♂)		
	#8	6,186	8 (5♀3♂)		
Cattle (n:4, 8.7%)	#31	8,596	1♂	*Rhipicephalus* spp.	10/75 (13.3%)
	#19	598	1♀		
	#11	247,466	7♂		
	#3	501,204	1♀		
Hyena (n:4, 8.7%)	#79	186	1♂	*Amblyomma* spp.	4/75 (5.3%)
	#68	156,604	1♂	*Rhipicephalus* spp.	
	#36	3,494	1♂		
	#12	1,182	1♂		
Plains Zebra (n:3, 6.5%)	#66	1,010	1♂	*Amblyomma* spp.	3/75 (4%)
	#40	3,698	1♂	*Rhipicephalus* spp.	
	#35	4,752	1♀		
Hartebeest (n:2, 4.3%)	#57	155,402	1♂	*Rhipicephalus* spp.	2/75 (2.6%)
	#54	1,013,892	1♂		
Wild dog (n:2, 4.3%)	#60	602012	3♀	*Rhipicephalus* spp.	4/75 (5.3%)
	#56	37,670	1♂		
Boran (n:1, 2.1%)	#30	23,186	4(2♀2♂)	*Rhipicephalus* spp.	4/75 (5.3%)
Impala (n:1, 2.1%)	#55	600	1♀	*Rhipicephalus* spp.	1/75 (1.3%)
Giraffe (n:1, 2.1%)	#20	52,486	1♂	*Rhipicephalus* spp.	1/75 (1.3%)
Leopard (n:1, 2.1%)	#81	439,608	1♀	*Rhipicephalus* spp.	1/75 (1.3%)
B. Rhino (n:1, 2.1%)	#21	92,750	1♂	*Rhipicephalus* spp.	1/75 (1.3%)
W. Rhino (n:1, 2.1%)	#72	74,580	1♂	*Rhipicephalus* spp.	1/75 (1.3%)
**Total: 46 pools**					**75 ticks**

### Metagenomic Sequencing and Molecular Testing

Tubes containing tick pools (1–8 ticks/pool) were dipped in liquid nitrogen and homogenized using beads. Nucleic acid purification was conducted using the RNeasy Mini Kit (Qiagen, Hilden, Germany), and complementary DNA (cDNA) synthesis with random hexamers was performed using the RevertAid First Strand cDNA Synthesis Kit (Thermo Fisher Scientific, Hennigsdorf, Germany), as per the manufacturer’s recommendations. Tick nucleic acid extraction and cDNA conversion were carried out at IPR laboratories in Kenya, and cDNAs were sent to WRBU (United States) for further testing.

The cDNA was quantified using Quant-iT OliGreen ssDNA Assay Kit (Thermofisher Scientific, MA, United States) on a Fluoroskan FL instrument (Thermofisher Scientific, MA, United States). Libraries were prepared using KAPA HyperPlus Kits (Roche, CA, United States), as recommended by the manufacturer, with fragmentation step of 20 min at 35*^o^*C, ligation step of 90 min, and 20 PCR cycles. Library quantification and quality control were performed using the TapeStation 4,200 Automated Electrophoresis instrument (Agilent Technologies, VA, United States). Excess adapter dimer and small fragments were removed using KAPA pure beads (Roche, CA, United States). Unbiased metagenomic sequencing was performed on the NovaSeq platform (Illumina, CA, United States) (PE 2 × 150) at the Walter Reed Army Institute of Research (WRAIR). Samples were run on one lane of an S4 flow cell with XP workflow designed to maximize the overall raw reads output. Read quality was assessed using fastqc (Wingett and Andrews, 2018), and adapter trimming and sequence filtering were performed using Trimmomatic (Bolger et al., 2014).

Jingmen tick virus (JMTV) screening was performed by a specific nested PCR assay, targeting the viral NS5-like protein located on the genome segment 1, as described previously (Yu et al., 2020). Amplified products were visualized on 1% agarose gels, stained with GelGreen nucleic acid stain (Biotium, California, United States), and visualized after electrophoresis in a SmartDoc 2.0 blue light imaging system (Accuris Instruments, New Jersey, United States). PCR products were purified with ExoSapIT Express (Applied Biosystems, California, United States) using the manufacturer’s instructions and prepared for sequencing with BigDye Terminator v3.1 Cycle Sequencing Kit (Applied Biosystems, California, United States), with the PCR primers used for the second nested PCR step for JMTV (Yu et al., 2020). Capillary sequencing was done on an ABI 3,730 automated sequencer (PE Applied BioSystems) at the Laboratories of Analytic Biology, Smithsonian Institution—National Museum of Natural History.

### Bioinformatics and Phylogenetic Analysis

Raw data from the Illumina NovaSeq sequencing runs were uploaded to the CZ-ID platform (formerly ID-Seq), a publicly accessible cloud-based storage and analysis platform for metagenomic pathogen detection (Kalantar et al., 2020), and were analyzed using the built-in pipeline (version 6.8). The pipeline is open source and incorporates various steps for validation, which include selected host, human and barcode adaptor removal, quality assessment, alignment, assembly, and taxonomic identification using the National Center for Biotechnology Information (NCBI) nucleotide and protein databases. Pathogens were considered as significant when ≥ 2 nucleotide reads or contigs were verified in the final output. Relative abundances were calculated in samples with bacterial reads ≥ 10^2^ and expressed as taxon read count/total bacteria read count. Reads and contigs from viruses, parasites, and tick-borne bacteria were manually reviewed with the taxon identity confirmed using BLAST (Altschul et al., 1990).

Viral reads were also recovered using the VirIdAl pipeline (Budkina et al., 2021). This pipeline merged paired-end reads to increase sequence length after which sequences were adapter-trimmed and quality-filtered using fastp (Chen et al., 2018). Sequences with a complexity of <30%, with an average PHRED quality score of <20, and with <36 bases were removed. Host sequences were also removed with Bowtie2 (Langmead and Salzberg, 2012), using the most closely related available tick reference genome (*Rhipicephalus sanguineus*, ASM1333969v1). Data were then clustered using vsearch (Rognes et al., 2016) to recover “centroid” sequences formed with a 0.9 identity threshold. These sequences then entered a two-step alignment process. First, sequences were blasted against both viral NT and NR databases at high sensitivity (*e*-value 10^–3^) to give “virus-like” sequence matches. These were then passed to a second step alignment to the complete NCBI NT and NR databases (*e*-value 10^–10^) for final classification. We used CZ-ID for the initial workup and VirIdAl on samples with detectable virus signal. Outputs from both pipelines were combined for producing final contigs, following duplicate removal.

Virus genome assemblies and Sanger sequencing data analyses were carried out using Geneious Prime version 2022.0.2^1^. Nucleotide and deduced amino acid sequence alignments and pairwise comparisons were generated using CLUSTAL W (Thompson et al., 1994). MEGA11 was used for estimating the optimal substitution model on individual alignments and to infer evolutionary history according to the Bayesian information criteria (Tamura et al., 2021).

The raw reads from tick pools tested in this study are freely available in https://idseq.net/ under the project name “KWS ticks” and in National Center for Biotechnology Information (NCBI) biosample database with the ID: SUB11147880.

## Results

A total of 75 adult ticks collected from 15 animal species were sequenced in 46 pools. Most of the pools (84.8%) comprised single ticks, whereas multiple ticks (c. 2–8 individuals) were screened in 7 of the 46 pools (15.2%) (Table 1). Majority of the samples comprised *Rhipicephalus* spp. (73/75, 97.3%) and *Amblyomma* spp. (2/75, 2.7%). Host sequence data were utilized to confirm the genus-level identification in the tick pools. The average raw reads count of 9,313,080 was recorded per sample, whereas it was noted as 190,915 following the low-quality read and host removal.

### Bacteria

Of the 46 pools sequenced, 35 tick pools were analyzed for pathogenic bacterial species and relative abundances of major phyla. Eleven pools were omitted from the analysis due to the relatively low number of total bacteria reads (<10^2^). Members of the phylum *Proteobacteria* were abundant in the bacterial composition of the tick pools, comprising 51.6–97.5% of the total bacterial reads in 17 (48.6%) pools, regardless of the host species (Supplementary Table 1). *Actinobacter* and *Firmicutes* species provided the major source of bacterial reads in 6 (17.1%) and 2 (5.7%) pools, respectively. Predominance of a single bacterial species (with reads constituting over 50%) within a phylum was observed in certain pools, namely, *Proteus mirabilis* (pools #6, #48, #52, #53, #56, and #60), *Clostridium botulinum* (pools #3, #69, and #84), *Coxiella burnetii* (pool #21), and *Enterobacter cloacae* (pool #78). Other than these instances, no particular tendency in relative bacterial abundances or host species was noted.

**FIGURE 2:**
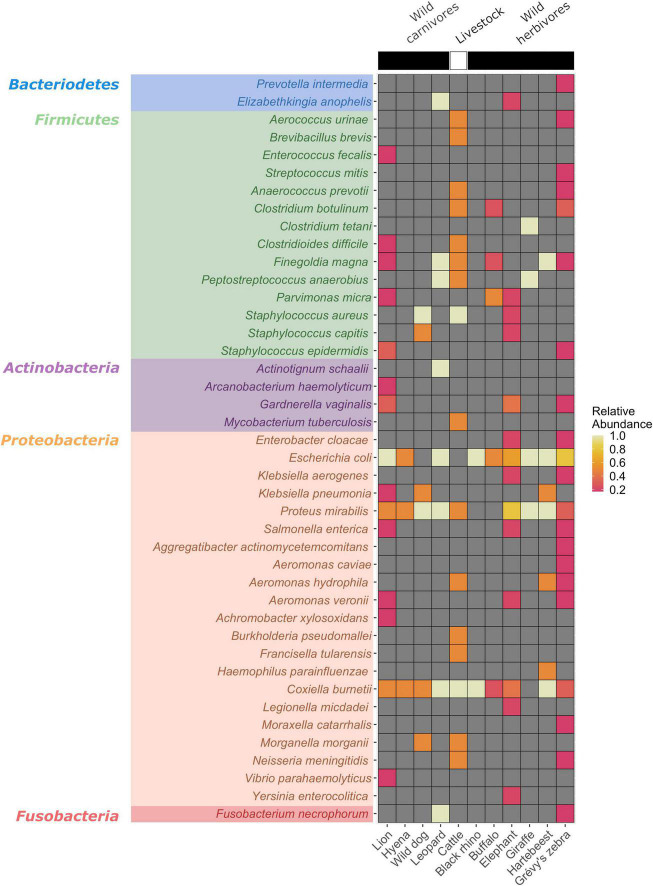
Heatmap of the detected bacterial pathogens according to the hosts. Pathogen detection rates were calculated as positive tick pools divided by the number of hosts examined.

A total of 56 bacterial species documented as human and/or animal pathogens were identified across the 35 pools examined (Figure 2). The most frequently detected pathogen was *E. coli* (22/35, 62.8%), followed by *P. mirabilis* (17/35, 48.5%) and *C. burnetii* (16/35, 45.7%). *C. burnetii* is the etiologic agent of Q fever, characterized by febrile and respiratory symptoms. Infections are zoonotic and are initiated by inhalation of particles contaminated by infected animals, most commonly sheep, goats, and cattle, with ticks implicated in transmission (Körner et al., 2021). *E. coli* and *P. mirabilis* are Gram-negative bacteria classified in the family *Enterobacteriaceae* alongside other potential pathogenic species in the genera *Klebsiella*, *Enterobacter*, *Citrobacter*, *Salmonella*, *Shigella*, and *Serratia.* Many are found in the normal gut flora of humans and animals, and others are widely distributed in soil and water. Commonly associated with infections of the urinary and gastrointestinal tracts, they can also produce severe and life-threatening, as well as nosocomial, infections (Mairi et al., 2018). Other Gram-negative bacteria of the genera *Achromobacter, Aeromonas, Aggregatibacter, Hemophilus, Morganella, Moraxella, Neisseria, Vibrio*, and *Yersinia* were detected in this dataset, with varying prevalences (Figure 2).

Of special note is the identification of *Francisella tularensis* and *Bacillus anthracis*, two zoonotic agents classified as Category A pathogens (National Institutes of Allergy and Infectious Diseases, 2018), owing to their significant public health threat and bioterrorism potential. *F. tularensis* was detected in 5 tick pools (5/35, 14.2%, read count range: 2–60) collected from cattle, lions, an elephant, and a Grevy’s zebra, whereas *B. anthracis* was identified in a single pool (1/35, 2.8%, read count: 22) collected from an elephant. A Gram-negative facultative intracellular bacterium, *F. tularensis*, causes tularemia, an anthropozoonosis that occurs *via* contact with wild animals, arthropod bites, or environment through respiratory, oral, or cutaneous routes (Maurin, 2020). Symptomatic disease may present with distinct clinical forms and can be mild or severe, depending on the entry route. As *Ixodidae* ticks are common arthropod vectors, detection of *F. tularensis* in tick pools indicates probable activity in the region. *B. anthracis* is an endospore-forming Gram-positive rod, causing anthrax in livestock and humans. In humans, cutaneous, respiratory, or gastrointestinal infections have been described, which can be fatal without proper treatment (Finke et al., 2020). A related bacteria, *Bacillus cereus*, mostly associated with gastrointestinal symptoms, was also detected (Figure 2).

We identified other *Firmicutes* such as *Burkholderia pseudomallei*, an opportunistic environmental bacterial pathogen of humans and animals, causing melioidosis (Limmathurotsakul et al., 2016). Moreover, pathogenic *Vibrio*, including *Vibrio cholerae*, were also detected. Facultative anaerobic cocci of the *Streptococcus* and *Staphylococcus* genera and anaerobic cocci and bacilli of various genera including *Clostridium* spp. were also observed. Most of the listed bacteria are ubiquitous in soil and gut microbiota of various animals as well as humans. Interestingly, *C. botulinum*, producer of the botulinum neurotoxin, was detected in 4 tick pools and emerged as the single predominant bacterial species in 3 of these pools, collected from cattle, buffalo, and Grevy’s zebra. Documented pathogens within the *Actinomycetota* phylum include *Corynebacterium*, *Gardnerella*, and *Mycobacterium* species. *Fusobacterium necrophorum*, classified in the phylum *Fusobacteriota*, is observed as the only pathogen outside the aforementioned bacterial phyla (Holm et al., 2016; Figure 2).

### Fungi

Eight fungal species listed as human pathogens were detected in 5 tick pools (10.8%) with low number of reads (range: 2-7). *Candida parapsilosis* was the most frequent pathogen (4.3%) identified in ticks collected from a hartebeest and a lion (Table 2). It is among the major causative agents of hospital-acquired invasive fungal disease, with severe outcomes in critically ill patients (Tóth et al., 2019). Not being an obligate pathogen, *C. parapsilosis* can be found in domestic animals, insects, soil, and marine environments, as well as a commensal of the human skin. Similarly, the remaining fungi are mostly opportunistic pathogens frequently found in the environment. They are associated with transcutaneous inoculation (chromoblastomycosis, *Cladophialophora* spp.) or diagnosed as opportunistic pathogens in immunocompromised hosts, with the exception of *Crypotococcus* spp., and are mainly disseminated by inhalation of the infectious particles from environmental sources (Gushiken et al., 2021).

**TABLE 2:** Overview of non-bacterial pathogens detected in tick pools.

	Species	Prevalence	Host^1^	Pool Code	Read Count
Fungi	*Aspergillus flavus*	1/46 (2.1%)	Hartebeest (1/2)	#54	4
	*Candida parapsilosis*	2/46 (4.3%)	Hartebeest (1/2)	#54	7
			Lion (1/7)	#23	2
	*Cladophialophora bantiana*	1/46 (2.1%)	Lion (1/7)	#10	2
	*Cladophialophora carrionii*	1/46 (2.1%)	Leopard (1/1)	#81	4
	*Cryptococcus neoformans*	1/46 (2.1%)	Leopard (1/1)	#81	2
	*Cryptococcus gattii*	1/46 (2.1%)	Hartebeest (1/2)	#54	2
	*Pneumocystis jirovecii*	1/46 (2.1%)	Lion (1/7)	#47	3
	*Rhizopus microsporus*	1/46 (2.1%)	Leopard (1/1)	#81	2
Parasites	*Brugia malayi*	2/46 (4.3%)	Leopard (1/1)	#81	2
			Lion (1/7)	#47	4
	*Dirofilaria immitis*	1/46 (2.1%)	Elephant (1/7)	#52	2
	*Encephalitozoon intestinalis*	1/46 (2.1%)	Lion (1/7)	#47	2
	*Entamoeba histolytica*	1/46 (2.1%)	Hyena (1/4)	#68	2
	*Loa loa*	1/46 (2.1%)	Elephant (1/7)	#52	2
	*Naegleria fowleri*	1/46 (2.1%)	Elephant (1/7)	#52	3
	*Plasmodium falciparum*	3/46 (6.5%)	Lion (3/7)	#43,#47,#53	8,2,4
	*Plasmodium malariae*	1/46 (2.1%)	Hartebeest (1/2)	#54	3
	*Plasmodium vivax*	1/46 (2.1%)	Hartebeest (1/2)	#54	2
	*Schistosoma haematobium*	1/46 (2.1%)	Cattle (1/4)	#3	2
	*Schistosoma mansoni*	4/46 (8.7%)	Hartebeest (2/2)	#54,#57	2,3
			Lion (1/7)	#47	5
			Elephant (1/7)	#52	4
	*Toxoplasma gondii*	2/46 (4.3%)	Leopard (1/1)	#81	2
			Wild dog (1/1)	#60	2
	*Trichinella spiralis*	6/46 (13%)	Lion (2/7)	#47,#53	4,736
			Elephant (1/7)	#52	54
			Leopard (1/1)	#81	4
			Grévy’s zebra (1/7)	#83	7
			Hartebeest (1/2)	#54	8
	*Trypanosoma cruzi*	3/46 (6.5%)	Grévy’s zebra (1/7)	#70	2
			Leopard (1/1)	#81	2
			Lion (1/7)	#47	2
Viruses	Human mastadenovirus C	10/46 (21.7%)	Grévy’s zebra (2/10)	#67,#83	4,6
			Leopard (1/10)	#81	22
			Elephant (3/10)	#6,#65,#71	2,2,4
			Lion (3/10)	#10,#22,#43	2,4,4
			Wild Dog (1/10)	#56	2
	Bovine alphaherpesvirus 5	4/46 (8.7%)	Buffalo (1/3)	#84	34
			Leopard (1/3)	#81	24
			Cattle (1/3)	#3	72
			Grévy’s zebra (1/3)	#69	3
	Epstein-Barr virus	1/46 (2.1%)	Lion (1/1)	#47	1660
	Jingmen tick virus	6/46 (13%)	Elephant (2/7)	#41,#52	2, n.a.
			Cattle (1/4)	#11	2
			Lion (1/7)	#10	2
			Hartebeest (1/2)	#54	n.a.
			Wild Dog (1/2)	#60	n.a.

*^1^Positive/total number of the species.*

*n.a.: not applicable due to detection by specific PCR.*

### Parasites

A total of 14 protozoan or nematode species listed as human pathogens were detected in 13 pools (28.2%), mostly in low to moderate number of reads (range: 2–736) (Table 2). The most frequent of these was *Trichinella spiralis*, detected in 6 pools (13%) collected from various hosts. It is a nematodal parasite of many carnivorous and omnivorous animals, where infections occur by ingestion of muscle tissue with encysted larvae (Ribicich et al., 2020). Human infections called trichinellosis or trichinosis are due to the consumption of undercooked meat. In most hosts, adult worms present in the intestine may continue to produce larvae for prolonged periods. Other than *Trichinella*, *Schistosoma mansoni, Trypanosoma cruzi*, and *Plasmodium* spp. were also detected, with prevalences of 6.5–8.7% (Table 2). However, co-detection of several parasites in particular pools [such as pool #52 from an elephant (*T. spiralis*, *S. mansoni*, *Naegleria fowleri*, *Loa loa*, *Dirofilaria immitis*) and pool #47 from a lion (*T. spiralis*, *S. mansoni*, *T. cruzi*, *Brugia malayi*, *Encephalitozoon intestinalis*)] strongly suggests an environmental origin for most of the identified pathogens. Moreover, human *Plasmodium falciparum* sequences detected most likely indicate prior feeding of the ticks on infected humans, evidencing the potential for cross-species zoonotic pathogen transfer from wildlife to humans *via* tick bites in African dryland ecosystems (Cable et al., 2017).

### Viruses

We recovered 4 viruses with significant human or veterinary health impact (Table 2). Human mastadenovirus C was most frequent viral pathogen, detected in 10 tick pools (10/46, 21.7%). Commonly referred as the adenoviruses, mastadenoviruses comprise a separate genus in the Adenoviridae family. They are non-enveloped viruses with double-stranded DNA genomes. Human mastadenovirus C is among the 51 currently described species of the genus, for which humans and other mammals such as bats, bovids, canine, equids, caprids and suids serve as natural hosts (Harrach et al., 2019). Symptomatic infections in humans may result in acute respiratory illness of variable severity, as well as conjunctivitis and gastroenteritis, which are usually mild.

Bovine alphaherpesvirus 5 (BHV-5) was detected in 4 tick pools (8.7%), including one collected from cattle (pool #3, Table 2). It is an enveloped double-stranded DNA virus classified in the *Varicellovirus* genus of the *Alphaherpesvirinae* subfamily. Similar to other alphaherpesviruses, BHV-5 establishes latency in the central nervous system of the exposed animals and is excreted in ocular, nasal and genital secretions upon reactivation (Del Médico Zajac et al., 2010). It causes severe meningoencephalitis in cattle, with high mortality in young calves. Sporadic cases and outbreaks have been reported in several countries in South America, Europe, and Asia. Another herpes virus, Epstein-Barr virus (EBV), was also detected in a tick pool collected from a lion (Table 2). It is another globally prevalent human herpesvirus and the causative agent of infectious mononucleosis. Originally discovered from African Burkitt’s lymphoma cells, EBV mainly infects B lymphocytes, may cause reactivating infections, and is associated with particular autoimmune syndromes (Houen and Trier, 2021).

**FIGURE 3:**
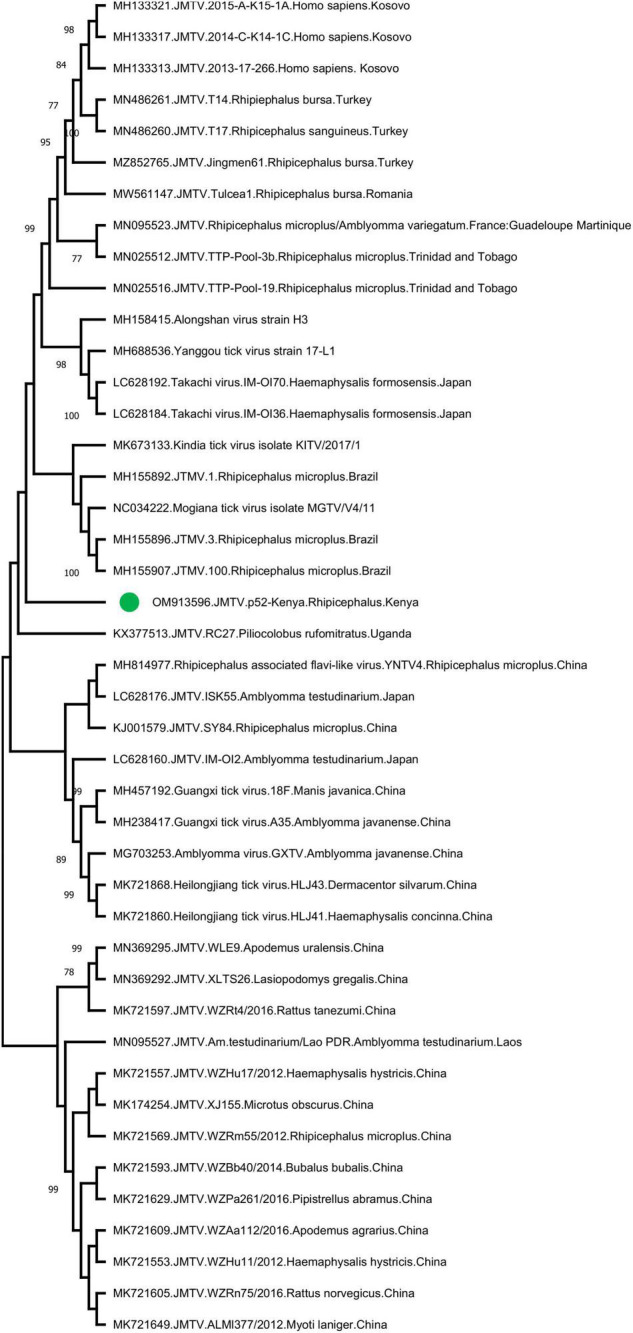
The maximum likelihood analysis of the Jingmen tick virus (JMTV) partial segment 1 sequences (366 nucleotides). The tree is constructed using a gamma-distributed Kimura 2-parameter model for 500 replications. JMTVs included in the analysis are indicated by GenBank accession number, name, isolate/strain identifier, host, and detection region. JMTV sequence characterized in this study is marked. Bootstrap values greater than 75 are displayed.

Jingmen tick virus is the only virus where tick-borne transmission appears as the main mode of spread in the study. JMTV sequences were detected in a total of 6 pools (13%) (Table 2). Metagenomic screening initially revealed 3 positive pools (from an elephant, cattle, and lion), whereas 3 additional pools were subsequently identified using JMTV-specific PCR amplification. Initially described from China, JMTV is the index of a new group of viruses (called the Jingmenvirus group) tentatively classified within *Flaviviridae* family (Temmam et al., 2019). JMTV possesses an RNA genome of 4 segments, where two segments encoding for the non-structural proteins are related to those from flaviviruses (Qin et al., 2014). It is reported as a human pathogen causing tick-borne infections presenting with mild to severe febrile diseases (Jia et al., 2019). We recovered a 366-nucleotide segment of the JMTV NS5-like protein-coding region by sequence analysis of the amplified product in pool #52 (GenBank accession: OM913596). The sequence displayed up to 94.62% nucleotide and 99.23% deduced amino acid identities to previously characterized JMTVs. Maximum likelihood analysis showed that the sequence is phylogenetically distinct from all previously described JMTV clades from various regions, as well as from related viruses such as Mogiana tick virus (Figure 3). The closest relative appeared as the JMTV-RC27, identified in a plasma sample of an eastern or Tana River red colobus monkey (*Piliocolobus rufomitratus*) from Uganda.

**FIGURE 4:**
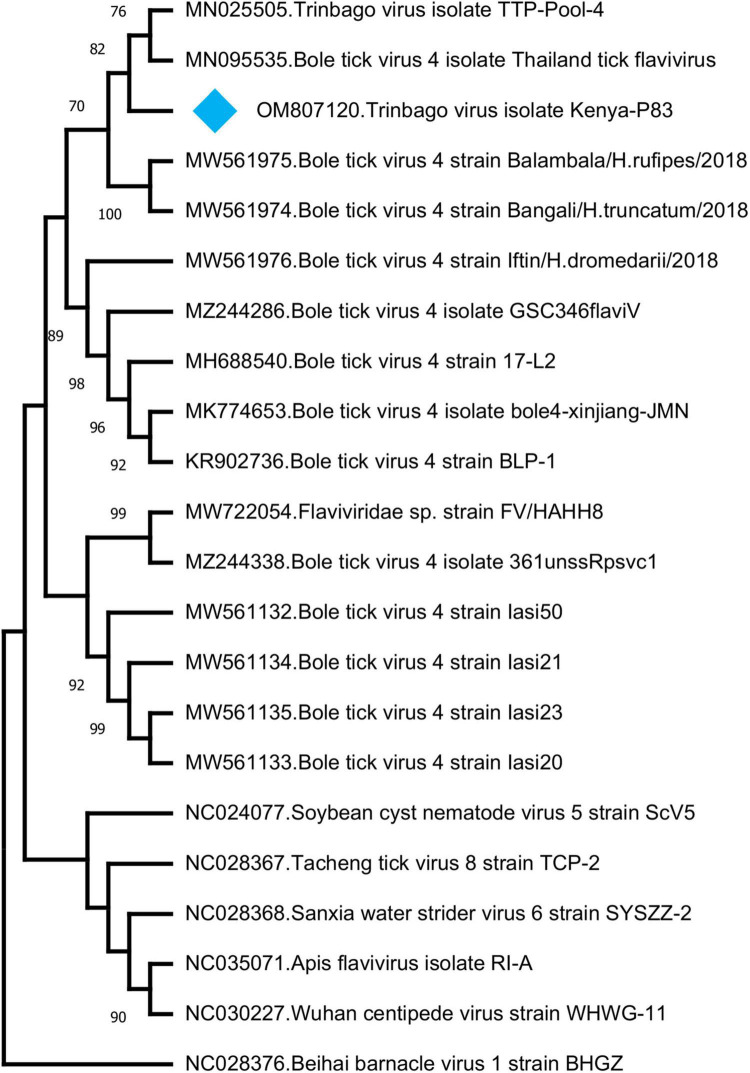
The maximum likelihood analysis of the Trinbago virus partial genome sequences (1,141 nucleotides). The tree is constructed using a gamma-distributed Kimura 2-parameter model for 500 replications. Viruses included in the analysis are indicated by GenBank accession number, name and isolate/strain identifier. Trinbago virus isolate Kenya-P83 characterized in this study is marked (GenBank accession: OM807120). Beihai barnacle virus 1 strain BHGZ is included as the outgroup.

In addition to those listed above, we identified other viruses not currently associated with disease in vertebrates. In 8 (17.3%) of the pools, sequences with the highest nucleotide and deduced amino acid identities to Trinbago virus (TBOV) were detected. TBOV is a recently described virus, distantly related to *Pestivirus* genus of the family *Flaviviridae* (Sameroff et al., 2019). The TBOV sequences identified in ticks vary in size and cover different parts of the viral genome without overlaps (Table 3 and Supplementary Table 2). The longest contig (1,141 base pairs), originating from pool 81 and covering approximately 10% of the genome, was used for phylogeny construction. In the maximum likelihood analysis, this sequence remained distinct and shared a common ancestor with the initial TBOV isolate (identified in *R. sanguineus* ticks from Trinidad and Tobago) and closely related Bole tick virus 4 isolate Thailand tick flavivirus (Figure 4).

**TABLE 3:** Viruses further detected in tick pools.

	Pool	Read Count	Host	Contig Length	Genome Location^1^	Similarity^1^	
						nt	aa
Trinbago virus	3	18	Cattle	338 bp	12823-13160	93.7%	97.3%
	11	14	Cattle	436 bp	12042-12477	91.9%	95.1%
	43	13	Lion	334 bp	10488-10821	87.4%	85.5%
	53	2	Lion	217 bp	12680-12896	71.4%	70.4%
	57	2	Hartebeest	230 bp	13839-14068	92.6%	94,7%
	70	3	Grévy’s zebra	246 bp	6414-6659	94.3%	95.0%
	83	3	Grévy’s zebra	470 bp	925-1394	85,1%	85.8%
	81	46	Leopard	1141 bp	4679-5819	89.8%	93.6%
Guarapuava tymovirus-like 1 virus	83	32	Grévy’s zebra	486 bp	4657-5142	79.2%	90.1%
	53	78	Lion	1802 bp	3564-5348	82.4%	91.6%
	52	2	Elephant	336 bp	3872-4207	81.2%	88.2%
	43	1238	Lion	5898 bp	172-6069	80.4%	89.7%

*^1^According to Trinbago virus isolate TTP-Pool-4 (MN025505) and Guarapuava tymovirus-like 1 isolate 3 (MH155881).*

*bp: base pairs, nt: nucleotide, aa: amino acid.*

We further identified Guarapuava tymovirus-like virus (GTLV) sequences in 4 (8.7%) pools and pairwise comparisons revealed the highest identities to GTLV-1 (Table 3 and Supplementary Table 3). GTLVs have been recently described in ticks and appear as highly divergent viruses within the order *Tymovirales* (Souza et al., 2018). Partially overlapping contigs and a complete coding sequence could be obtained from the pools (tentatively named as GTLV-1 isolate Kenya-P43), with no further effort to confirm the non-coding 5′ and 3′ ends of the viral genome.

**FIGURE 5:**
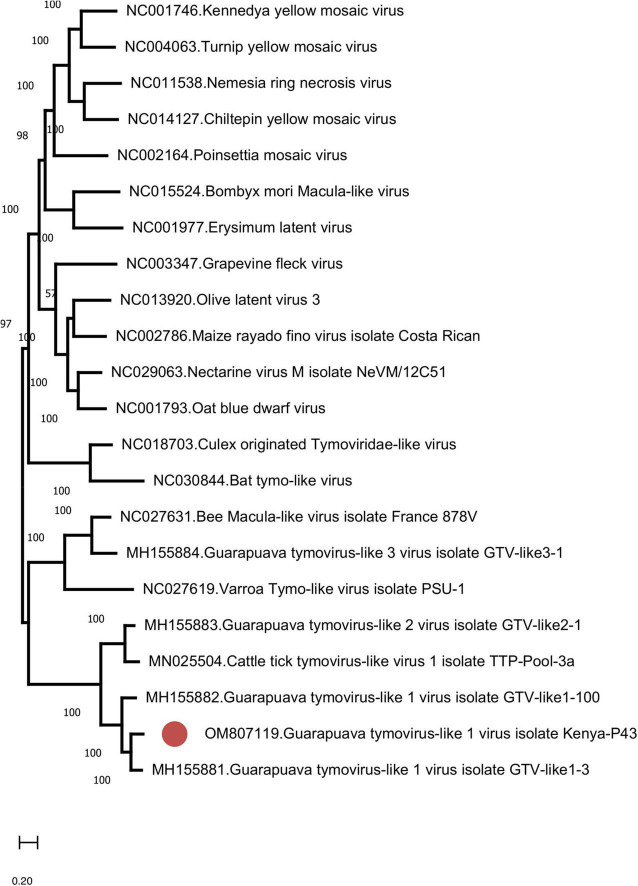
The maximum likelihood analysis of the Guarapuava tymovirus-like virus ORF sequences (5,898 nucleotides). The tree is constructed using the general time reversible (GTR) model, gamma distributed with invariant sites (G + I) model for 500 replications. Guarapuava tymovirus-like 1 virus isolate Kenya-P43 characterized in this study is marked (GenBank accession: OM807119). Bootstrap values greater than 50 are displayed.

Similar to GTLVs, two open reading frames (ORFs) are recognized in the GTLV-1 isolate Kenya-P43 genome, encoding for the ORF1 polyprotein (nucleotides 1–5031) and viral capsid (nucleotides 5,041–5,898) (Souza et al., 2018). Analysis of the deduced amino acid sequences of these viral proteins revealed several functional motifs pertaining to intracellular virus replication (Table 4). Phylogeny reconstruction revealed clustering of GTLV-1 and 2 viruses and a distinct separation of GTLV-1 isolate Kenya-P4 (Figure 5).

**TABLE 4:** Functional motifs in the Guarapuava tymovirus-like 1 virus isolate Kenya-P43 genome.

Motif	Domain Accession	Location^1^
Viral methyltransferase	pfam01660	54-326
UL36 large tegument protein	PHA03247	458-623
Tymovirus endopeptidase	pfam05381	658-742
Viral helicase	pfam01443	836-1071
RNA-dependent RNA polymerase	pfam00978	1364-1585
Tymovirus capsid protein	pfam00983	1784-1959

*^1^According to polyprotein deduced amino acid sequence (OM807119).*

## Discussion

Our cross-sectional metagenomic investigation of ticks collected from wildlife species revealed a broad spectrum of bacterial, fungal, parasitic, and viral pathogens. We identified tick-borne pathogens *F. tularemia* and JMTV in 14.2 and 13% of the tick pools, respectively, with both agents observed in ticks collected from wild animals and cattle. Currently, tularemia is considered a global re-emerging zoonotic disease and *F. tularemia* as a potential agent of biological warfare, due to its ease of aerosol dissemination, low infectious dose, and potential fatal outcomes (Maurin, 2015). Other than the evidence of probable exposure in patients with undifferentiated febrile illness (Njeru et al., 2017), *F. tularemia* has not previously been reported from Kenya. Our findings indicate that it is present in potential vectors in livestock and wildlife, requiring further screening and specific diagnosis in cases presenting with compatible symptoms.

The JMTV is a recently described virus associated with tick-borne diseases in humans. Initially discovered in *Rhipicephalus microplus* ticks from China, the virus has been shown to be distributed in many countries in Eurasia and the Americas, and also in a variety of non-tick hosts including cattle, rodents, bats, and humans (Guo et al., 2020). Human infections were initially documented in Crimean-Congo hemorrhagic fever cases from Kosovo (Emmerich et al., 2018) and later confirmed in China (Jia et al., 2019). Moreover, genetically related viruses with similar genomic structure—some also associated with human disease (Alongshan virus) have been described—are collectively referred to as the Jingmen virus group (Shi et al., 2015; Ladner et al., 2016; Kholodilov et al., 2020, 2021). The first documentation of a Jingmen virus in the African continent was a JMTV variant genome sequence (RC27) in Uganda, detected in plasma collected from a red colobus monkey (*P. rufomitratus*), a critically endangered primate (Ladner et al., 2016). Hereby, we report the detection of JMTV in ticks from Kenya as well as from Africa. Interestingly, phylogenetic reconstructions based on a relatively short segment of the virus genome showed significant divergence from previously known JMTVs and related viruses, likely to represent variations due to geographical segregation (Figure 3). Further screening and sequencing efforts will elucidate JMTV genome diversity in Kenya as well as the African continent. As of this writing, JMTV was reported in ticks from two other pastoralist-dominated areas (Ogola et al., 2022).

We further detected another pathogenic bacteria, *C. burnetii* in 45.7% of the tick pools and in all host species except for a giraffe (Figure 2). It is also observed as one of the frequently identified bacteria in our tick cohort. *C. burnetii* is known to display a wide host range and can replicate in many mammalian, avian, and reptilian species, as well as arthropods (Yon et al., 2019). However, the role of wildlife in the Q fever epidemiology in livestock and humans is yet to be established (Körner et al., 2020). Moreover, despite evidence for vector potential, the role of ticks in transmission still remains controversial. The presence of *C. burnetii* in Kenya is well established, with detections in various cohorts of ticks and their hosts, including those from wildlife and at the wildlife-livestock interface (Knobel et al., 2013; Ndeereh et al., 2017; Koka et al., 2018; Getange et al., 2021). It demonstrates a widespread circulation in the country and is likely to be a causative agent of undiagnosed febrile disease in pastoral communities. Our findings confirm previous reports and suggest wildlife-livestock interactions to contribute further to *C. burnetii* transmission.

Other bacterial agents with high pathogenicity identified in the study include *B. anthracis* and *C. botulinum*. Anthrax is among the top priority zoonotic diseases in Kenya (Nderitu et al., 2021). Infections have been reported in over 30% of Kenyan wildlife conservancies, with the majority occurring in dry seasons and primarily affecting herbivore species including buffalo, black and white rhinos, and elephants (Gachohi et al., 2019). In this study, we detected *B. anthracis* in a single tick (2.8%) collected feeding on an elephant, demonstrating ongoing activity in wildlife. Botulinum toxin produced by the anaerobic rod *C. botulinum* causes severe poisoning, frequently occurring due to oral intake. *C. botulinum* is ubiquitously found in soil and aquatic sediments that serve as an environment for sporulation (Espelund and Klaveness, 2014). Botulinum toxin produced by *C. botulinum* can intoxicate and kill various animal species upon entering their food webs. It has been detected in soil specimens and has caused documented outbreaks in Kenya (Smith et al., 1979; Nightingale and Ayim, 1980; Yamakawa et al., 1990). We identified *C. botulinum* in a total of 4 pools (8.7%) collected from cattle, buffalo, and endangered Grevy’s zebra. In this study, the source is likely to be environmental in origin. However, the presence of *C. botulinum* as the single bacterial species in 3 pools suggests that it can readily predominate and pose a threat to human and animal health without particular precautions.

In addition to the tick-borne or highly pathogenic species discussed above, we detected many species of bacteria in the tick pools, where *E. coli* and *P. mirabilis* constitute the most frequent species, identified in 62.8% and 48.5% of the pools, respectively. Furthermore, several other species *of Enterobacteriaceae*, *Staphylococci*, *Streptococci*, and *Clostridia* spp. other than *C. botulinum* were present in tick pools (Figure 2). These bacteria are either ubiquitously found in soil or are present in gut or skin microbiota of several host species including humans, some being consistently shed in feces. Although they are widespread in nature, they can be opportunistic pathogens in specific hosts in various settings (such as nosocomial infections) (Lax and Gilbert, 2015) or can cause zoonotic infections (such as *B. pseudomallei*). Similarly, we identified several fungi including *Aspergillus*, *Candida*, *Cladophialophora*, *Cryptococcus*, Pneumocystis, and *Rhizopus* species, found widespread in nature, that mostly cause opportunistic infections. However, *Cryptococcus neoformans and Cryptococcus gattii*, detected in the study in 4.2% of the pools, are documented to produce severe infections in immunocompetent individuals as well (May et al., 2016; Hsu et al., 2022). Infections and genotypes of *Cryptococcus* species have been previously reported from Kenya (Kangogo et al., 2015).

Our metagenomic analysis displayed several medically important parasites, most of which are endemic in the region (Table 2). The vector-borne filarial pathogens (*B. malayi, D. immitis*, and *L. Loa*) and protozoa (*Plasmodium* spp. and *T. cruzi*) detected in ticks probably represent blood meals from infected hosts. The environmental and water-/food-borne pathogens *Entamoeba histolytica, Encephalitozoon intestinalis, Naegleria fowleri, Schistosoma* spp., *Toxoplasma gondii*, and *Trichinella spiralis* were detected, which indicate, along with the diversity of parasites detected in single ticks, contamination from environmental sources. These findings demonstrate a marked potential for wildlife-livestock-human pathogen transfer. This is also supported by the frequent detection of adenovirus and herpesvirus that cause human or cattle infections (Table 2).

We further described two recently characterized viruses with currently unknown health impact, namely, GTLV-1 and TBOV, and documented their first reporting from the African continent. These viruses were prevalent in ticks with co-detection in three pools (Table 3). TBOV was initially discovered in Trinidad and Tobago and was present in all *Rhipicephalus* and *Amblyomma* spp. screened in the region (Sameroff et al., 2019). The TBOV genome shares significant similarities to Bole tick virus 4, a tick-associated virus discovered in China (Shi et al., 2015). Particular non-structural proteins from both viruses share similarities to Pestiviruses in the family *Flaviviridae*, albeit forming a distinct phylogenetic clade suggesting a novel genus (Sameroff et al., 2019). TBOV has been documented in a wide range of tick species, suggesting acquisition from an unknown vertebrate host. Since *Flaviviridae* family includes several tick-borne viral pathogens, potential association of TBOV with vertebrate infections or its impact on vector capacity for tick-borne flaviviruses requires further investigation. Virome analyses performed in ticks collected from camels in Kenya showed Bole tick virus 4 in three *Hyalomma* tick species and virus exposure in hosts (Zhang et al., 2021). We further detected a local GTLV-1 isolate and analyzed its complete coding region (Table 4). Similar to TBOV, GTLVs including GTLV 1, 2, and 3 are novel viruses described recently in *R. microplus* ticks from Brazil, tentatively named due to their limited genome similarities to members of the order *Tymovirales* (Souza et al., 2018). Tymoviruses are plant viruses associated with mosaic disease where arthropods facilitate spread as mechanical vectors (Lefkowitz et al., 2018). Due to their divergent genomes, GTLVs have been proposed as a novel family, with currently unknown pathogenicity in invertebrate hosts (Souza et al., 2018).

Finally, particular limitations or shortcomings of the study must be addressed. Due to the sampling approach and storage conditions, we were unable to provide a species-level identification of ticks. Moreover, the sequencing runs produced a relatively lower number of reads in the tick pools, which is probably due to ethanol storage, a frequently used approach to preserve entomological specimens. For metagenomic investigations, we used a straightforward cDNA-based approach, without prior treatment to enrich particular targets such as viruses. Nevertheless, given the breadth of microbial pathogens detected, our approach has been successful even in ethanol-stored specimens, enabling the identification of many human and animal pathogens, some having been documented for the first time in the region. We also observed ticks as a promising sentinel to monitor pathogen circulation in domestic-wildlife interfaces in the Kenyan landscape. Due to their natural life cycle that may involve several host animals, they provide information not only on tick-borne agents but from hosts and the environment as well. Despite the costs and requirement of a significantly equipped laboratory infrastructure and trained personnel, metagenome-based investigations are capable of producing crucial information on the identification of circulating pathogens, prioritization of targets for surveillance, or management of mitigation efforts.

In conclusion, we detected several microbial pathogens by a metagenomic approach in ticks collected from animals at the livestock-wildlife interfaces in Kenya. Tick-borne pathogens JMTV and *F. tularensis* were documented in Kenya, as well TBOV and GTLV-1, with currently unexplored impact on vertebrate pathogens.

## Data Availability Statement

The raw reads from tick pools tested in this study are available in https://idseq.net/ under the project name “KWS ticks”, and in the National Library of Medicine - National Center for Biotechnology Information (NCBI, https://www.ncbi.nlm.nih.gov) Biosample and Sequence Read Archive (SRA) under accession numbers 28626882–28626931.

## Author Contributions

KE: laboratory analysis, data analysis and interpretation, and manuscript drafting. MM: wildlife specimen collection, transport, and initial processing. BB: laboratory analysis and data analysis and interpretation. SJ: laboratory analysis and data analysis. LC-Q: laboratory analysis and data analysis. JK: wildlife specimen collection, transport, and initial processing. SaM: wildlife specimen collection, transport, and initial processing. IA: wildlife specimen collection, transport, and initial processing. EC: wildlife specimen collection, transport, and initial processing. FG: wildlife specimen collection, transport, and initial processing. PO: wildlife specimen collection, transport, and initial processing. SuM: study design. DZ: study design and data interpretation. Y-ML: study design and conception, data interpretation, and manuscript drafting. All authors contributed, read and approved the submitted manuscript.

## Conflict of Interest

MM was employed by Kenya Wildlife Services Corporation. The remaining authors declare that the research was conducted in the absence of any commercial or financial relationships that could be construed as a potential conflict of interest.

## Publisher’s Note

All claims expressed in this article are solely those of the authors and do not necessarily represent those of their affiliated organizations, or those of the publisher, the editors and the reviewers. Any product that may be evaluated in this article, or claim that may be made by its manufacturer, is not guaranteed or endorsed by the publisher.

## Footnotes

1
https://www.geneious.com
