# Supplementary material for: Metagenomic Investigation of Ticks From Kenyan Wildlife Reveals Diverse Microbial Pathogens and New Country Pathogen Records
Source: Front Microbiol. 2022 Jul 1;13:932224. doi: 10.3389/fmicb.2022.932224 (PMC9283121; doi:10.3389/fmicb.2022.932224)
Supplement: Supplementary file 2 [file Data_Sheet_1.PDF]

Supplement 2: Alignment of the Trimbago virus sequences detected in pools #3, #11, #43, #53, #57, #70, #81 and #83. Positions are given according to the Trinbago virus isolate TTP-Pool-4 (MN025505).

```
                       910       920       930       940       950       960       970       980       990      1000
                ....|....|....|....|....|....|....|....|....|....|....|....|....|....|....|....|....|....|....|....|
Trinbago virus  GCCAGAAGGCGAACGGCTACGGCCGTCGCCCTTCTCCCTCTCATCGCCAATGCCGACGCAATCTGGACCACCAGAACGGTCGGCACGAACATGATCAACG
p3              ----------------------------------------------------------------------------------------------------
p11             ----------------------------------------------------------------------------------------------------
p43             ----------------------------------------------------------------------------------------------------
p53             ----------------------------------------------------------------------------------------------------
p57             ----------------------------------------------------------------------------------------------------
p70             ----------------------------------------------------------------------------------------------------
p81             ------------------------GTCGCCCTTCTCCCTCTCATCGCCAATGCCAACGCAATCTGGACCACCAGAACAGTCGGCACGAACATGATCAACG
p83             ----------------------------------------------------------------------------------------------------

                      1010      1020      1030      1040      1050      1060      1070      1080      1090      1100
                ....|....|....|....|....|....|....|....|....|....|....|....|....|....|....|....|....|....|....|....|
Trinbago virus  ACAATGGCACCCGCGATGCCATTGAGGTTACCGGCGAGGAGGTGGAATGGCACTGGTGGCCGTTCACCTGCTGCATACAGATTGACGACCAGGAGGCCCA
p3              ----------------------------------------------------------------------------------------------------
p11             ----------------------------------------------------------------------------------------------------
p43             ----------------------------------------------------------------------------------------------------
p53             ----------------------------------------------------------------------------------------------------
p57             ----------------------------------------------------------------------------------------------------
p70             ----------------------------------------------------------------------------------------------------
p81             ACAATGGCACCCGCGATGCCACTGAGGTCACCGGCGAGGCGGAAGATTGGCACTGGTGGCCTTTCACCTGCTGCATCCAGGTGGACGACCAGGAGGCTCA
p83             ----------------------------------------------------------------------------------------------------

                      1110      1120      1130      1140      1150      1160      1170      1180      1190      1200
                ....|....|....|....|....|....|....|....|....|....|....|....|....|....|....|....|....|....|....|....|
Trinbago virus  GACTGAACTAACTGGCGAGGAGTGGCTCACAACCCATATCTCCTCAGTCGAGCCCAGGGGCGTTGATTGGGCGCTTGACCCCAATGACTTGACCCCTAAC
p3              ----------------------------------------------------------------------------------------------------
p11             ----------------------------------------------------------------------------------------------------
p43             ----------------------------------------------------------------------------------------------------
p53             ----------------------------------------------------------------------------------------------------
p57             ----------------------------------------------------------------------------------------------------
p70             ----------------------------------------------------------------------------------------------------
p81             AACTGAACTCACTGGTGAGGCATGGCTCACGACCCATGTCTCTGCGGTGGAGGCAATGGGTGTCGACTGGGCGCTCGACCCAAACGACCTCACTCCCAAC
p83             ----------------------------------------------------------------------------------------------------

                      1210      1220      1230      1240      1250      1260      1270      1280      1290      1300
                ....|....|....|....|....|....|....|....|....|....|....|....|....|....|....|....|....|....|....|....|
Trinbago virus  ACCCCTGACGTCGCCTTCCAAGGCCAGGGGCGGGCACTGGCCAGCATTGTCCCCGAACGGCTCCAGGAAGAAGAGCCGCAGCCCGGACCCTCCAACCAGG
p3              ----------------------------------------------------------------------------------------------------
p11             ----------------------------------------------------------------------------------------------------
p43             ----------------------------------------------------------------------------------------------------
p53             ----------------------------------------------------------------------------------------------------
p57             ----------------------------------------------------------------------------------------------------
p70             ----------------------------------------------------------------------------------------------------
p81             ACCCCGGATGTGGCGTTTCAGGGGCAAGGGCAGTCACTCGCCAGCATCGTCCCTGAGCGGCTCCCAGAAGAAGAGCCGCAGTCCGGGCCCCCCACCCAGC
p83             ----------------------------------------------------------------------------------------------------
```

```
                      1310      1320      1330      1340      1350      1360      1370      1380      1390      1400
                ....|....|....|....|....|....|....|....|....|....|....|....|....|....|....|....|....|....|....|....|
Trinbago virus  AAACCTGCTCACAGCAGGGGAAGAAAAAGGACCCCCCATCCCAAGATGACGACGATAGCGACTCAGGCGCTGCCGGCCGCGCACCTCCTCCCCCCTCGAC
p3              ----------------------------------------------------------------------------------------------------
p11             ----------------------------------------------------------------------------------------------------
p43             ----------------------------------------------------------------------------------------------------
p53             ----------------------------------------------------------------------------------------------------
p57             ----------------------------------------------------------------------------------------------------
p70             ----------------------------------------------------------------------------------------------------
p81             AAACTTGCTCACAGCAGGGGAAGGAAAAGGACCCCCCCTCTCAAGATGACGACGACAGCGACTCAGATGCTGCCGGCCGGGCCCCTTCTCGCTC------
p83             ----------------------------------------------------------------------------------------------------
                      4610      4620      4630      4640      4650      4660      4670      4680      4690      4700
                ....|....|....|....|....|....|....|....|....|....|....|....|....|....|....|....|....|....|....|....|
Trinbago virus  TCCCAGAACGGTATGCCAACACCCCCGACGTCATCGCACACCTGCAAGACCTCTCAGACCTCTCGGGGGGAAAGAAAGGCACTTGCTTCGACAAGTGCAC
p3              ----------------------------------------------------------------------------------------------------
p11             ----------------------------------------------------------------------------------------------------
p43             ----------------------------------------------------------------------------------------------------
p53             ----------------------------------------------------------------------------------------------------
p57             ----------------------------------------------------------------------------------------------------
p70             ----------------------------------------------------------------------------------------------------
p81             ----------------------------------------------------------------------------------------------------
p83             ------------------------------------------------------------------------------GCACTTGTCGACGCAAGTGCCC
                      4710      4720      4730      4740      4750      4760      4770      4780      4790      4800
                ....|....|....|....|....|....|....|....|....|....|....|....|....|....|....|....|....|....|....|....|
Trinbago virus  CCAGACCAACCCCAACGACGACGAATTCCGTTGGTGTTGTGCAGGACGTAGCGGCACGCTCAACGAGCGCTGGACCCTCGTCAATGAAACCGTCTGCACA
p3              ----------------------------------------------------------------------------------------------------
p11             ----------------------------------------------------------------------------------------------------
p43             ----------------------------------------------------------------------------------------------------
p53             ----------------------------------------------------------------------------------------------------
p57             ----------------------------------------------------------------------------------------------------
p70             ----------------------------------------------------------------------------------------------------
p81             ----------------------------------------------------------------------------------------------------
p83             GGCGACCACCCCCAACGATGACGAGTTCCGTTGGTGTTGCGCAGGACGTAGCGGCACGCTCAATGAGCGCTGGACTCTCGTCAATGAAACCGTCTGTACA

                      4810      4820      4830      4840      4850      4860      4870      4880      4890      4900
                ....|....|....|....|....|....|....|....|....|....|....|....|....|....|....|....|....|....|....|....|
Trinbago virus  ACAGGCGGCACCTACTGTGGCTTCACCATTAACCCCAAGTGGGTGACAAAAAAGAATGTCATCTACGACGAGGCCACCGAACGCGAAACAGCCCGCCGCA
p3              ----------------------------------------------------------------------------------------------------
p11             ----------------------------------------------------------------------------------------------------
p43             ----------------------------------------------------------------------------------------------------
p53             ----------------------------------------------------------------------------------------------------
p57             ----------------------------------------------------------------------------------------------------
p70             ----------------------------------------------------------------------------------------------------
p81             ----------------------------------------------------------------------------------------------------
p83             ACGGGCGGCACCTACTGTGGCTTCACCATCAACCCTAAGTGGGTGACAAAAAAGAACGTCATTTATGATGAGGCCACCGAACGCGAGCCAGCCCGCCGCA

                      4910      4920      4930      4940      4950      4960      4970      4980      4990      5000
                ....|....|....|....|....|....|....|....|....|....|....|....|....|....|....|....|....|....|....|....|
Trinbago virus  TCGCCATGGCTGGCATCTATGGCGAGGATGAGCTCCAACCCGGCGGAGACGAAGACACCCTTGCGGTGCGAGGAGCCCTGGCCGCCTTCACCACCAGGAA
p3              ----------------------------------------------------------------------------------------------------
p11             ----------------------------------------------------------------------------------------------------
p43             ----------------------------------------------------------------------------------------------------
p53             ----------------------------------------------------------------------------------------------------
p57             ----------------------------------------------------------------------------------------------------
p70             ----------------------------------------------------------------------------------------------------
p81             ----------------------------------------------------------------------------------------------------
p83             TCGCCATGGCAGGCATCTATGGCGAGGATGAACTCCAACCCGGCGGAGACGAGGACACCCTTGCGGTGCGAGGAGCCCTGGCCGCCTTCACCACCAGGAA
```

```
                       5010      5020      5030      5040      5050      5060      5070      5080      5090      5100
               ....|....|....|....|....|....|....|....|....|....|....|....|....|....|....|....|....|....|....|....|
Trinbago virus GAGCGCGCCTTACTTAGGAGCCAGTGTCGACATTGGTTTTCTACGCTCCATCGCCGCTAAGATCGGCTTCGATGGCTTCTTCAAAGAAGCCGACATCAAC
p3             ----------------------------------------------------------------------------------------------------
p11            ----------------------------------------------------------------------------------------------------
p43            ----------------------------------------------------------------------------------------------------
p53            ----------------------------------------------------------------------------------------------------
p57            ----------------------------------------------------------------------------------------------------
p70            ----------------------------------------------------------------------------------------------------
p81            ----------------------------------------------------------------------------------------------------
p83            GAGCGCGCCGTACTTGGCAGCCAGTGTTGACATCGCCTTTCTTCGCTCCATCGCCGCCAAGATCTGTTTTGACGGCTTCTTTAAAGAGGCCGACATCAAC
                       5110      5120      5130      5140      5150      5160      5170      5180      5190      5200
               ....|....|....|....|....|....|....|....|....|....|....|....|....|....|....|....|....|....|....|....|
Trinbago virus TTTGCCGCCCCAGCTATCTTCCAGGTGCAGACCAACACGGGCTGCACAAACACCGGCTTCTGCTTCCAACACCGCGGAGCCGTTTTTACAAACTACCACG
p3             ----------------------------------------------------------------------------------------------------
p11            ----------------------------------------------------------------------------------------------------
p43            ----------------------------------------------------------------------------------------------------
p53            ----------------------------------------------------------------------------------------------------
p57            ----------------------------------------------------------------------------------------------------
p70            ----------------------------------------------------------------------------------------------------
p81            ----------------------------------------------------------------------------------------------------
p83            TTCGCCGCCCCCGCCATCTTTCAGGTGCAGACTAACACCGGCTGCACCAACACCGGCTACTGCTTCCACCACCGCGGAGCCTTCTTCACCAACTACCCCG
                       5210      5220      5230      5240      5250      5260      5270      5280      5290      5300
               ....|....|....|....|....|....|....|....|....|....|....|....|....|....|....|....|....|....|....|....|
Trinbago virus TCACAGAGGGCAGACCCCTCGTCCTACCGACTGTGTCCAACGAGGGCAAGGTGGATTACTCGCTGATCCGCTGCGTCTTCAAAAATGAGAAAGACGATCT
p3             ----------------------------------------------------------------------------------------------------
p11            ----------------------------------------------------------------------------------------------------
p43            ----------------------------------------------------------------------------------------------------
p53            ----------------------------------------------------------------------------------------------------
p57            ----------------------------------------------------------------------------------------------------
p70            ----------------------------------------------------------------------------------------------------
p81            ----------------------------------------------------------------------------------------------------
p83            TTACAGAGGGCAGACCCCTCGTCCTCCCCACCGTGTCCAACGAGGGCAAGGTGGATTACTCGTTAATCCGCTGTGTCTTCAAAAATGAGAAAGACGATCT

                       5310      5320      5330      5340      5350      5360      5370      5380      5390      5400
               ....|....|....|....|....|....|....|....|....|....|....|....|....|....|....|....|....|....|....|....|
Trinbago virus GGCTATCTACGGAAAGTCAGTCGCCCTCGACACCCCTGACATCGGGGAAGTGGGCATCGTCGTCAATCCCGCAGCACAACAGGCCATTGTGCTGCGTGTG
p3             ----------------------------------------------------------------------------------------------------
p11            ----------------------------------------------------------------------------------------------------
p43            ----------------------------------------------------------------------------------------------------
p53            ----------------------------------------------------------------------------------------------------
p57            ----------------------------------------------------------------------------------------------------
p70            ----------------------------------------------------------------------------------------------------
p81            ----------------------------------------------------------------------------------------------------
p83            GGCTATCTACGGAAAGTCAGTCGCCCTGGACACCCCCGACATGGGGGAGGTCGGCATCGTCGTTAACCCCGCAGCACAACAGGCCATCGTGCTGCGTGTG

                       5410      5420      5430      5440      5450      5460      5470      5480      5490      5500
               ....|....|....|....|....|....|....|....|....|....|....|....|....|....|....|....|....|....|....|....|
Trinbago virus AAAGGACCCACAGCAGGCCACGAAGCGACCCACAGCCGCCTCGACCGGTTTGTGTTCACCCAGAACTATGACGGACAAGTGCAGGCCGTAGAGTACAACA
p3             ----------------------------------------------------------------------------------------------------
p11            ----------------------------------------------------------------------------------------------------
p43            ----------------------------------------------------------------------------------------------------
p53            ----------------------------------------------------------------------------------------------------
p57            ----------------------------------------------------------------------------------------------------
p70            ----------------------------------------------------------------------------------------------------
p81            ----------------------------------------------------------------------------------------------------
p83            AAAGGACCCACAGCAGGCCATGAAGCGACCCACAGCCGGCTCGATCGGTTCGTGTTTACCCAAAACTATGACGGACAAGTGCAGGCCGTAGAGTACAATA
```

```
                       5510      5520      5530      5540      5550      5560      5570      5580      5590      5600
               ....|....|....|....|....|....|....|....|....|....|....|....|....|....|....|....|....|....|....|....|
Trinbago virus AGTACTCTCTCTACGGATGGAGCGGCCTGCCCATCATTTCCATTTCCTCCGGCCAGCCCATCGGCATATACGGCAAGATCGGGGTAGAGGACGGCAAGAA
p3             ----------------------------------------------------------------------------------------------------
p11            ----------------------------------------------------------------------------------------------------
p43            ----------------------------------------------------------------------------------------------------
p53            ----------------------------------------------------------------------------------------------------
p57            ----------------------------------------------------------------------------------------------------
p70            ----------------------------------------------------------------------------------------------------
p81            ----------------------------------------------------------------------------------------------------
p83            AGTACTCACTCTACGGATGGAGCGGCCTGCCCATCATTTCCATTTCCTCCGGCCAGCCTATTGGCATCTACGGCAAGATCGGGGTAGAGGACGGCAAGAA

                       5610      5620      5630      5640      5650      5660      5670      5680      5690      5700
               ....|....|....|....|....|....|....|....|....|....|....|....|....|....|....|....|....|....|....|....|
Trinbago virus GAACGTCGTACTCAGCCCCCAGACAAGTGGTCTAGTGCGCACAGTTGATTGGCGCAACCAAGCACGTCAACTTCTGGATGGCCCGCTCGAGATCCAGAAC
p3             ----------------------------------------------------------------------------------------------------
p11            ----------------------------------------------------------------------------------------------------
p43            ----------------------------------------------------------------------------------------------------
p53            ----------------------------------------------------------------------------------------------------
p57            ----------------------------------------------------------------------------------------------------
p70            ----------------------------------------------------------------------------------------------------
p81            ----------------------------------------------------------------------------------------------------
p83            GAACGTCGTGCGCACCCCACCGACACCTGAACGCGGACGCACGCTAGACTGGCGCAACCAGGCGCGTCAGCTTTTGGACGGCCCGCTTGAGATCCAAAAC

                       5710      5720      5730      5740      5750      5760      5770      5780      5790      5800
               ....|....|....|....|....|....|....|....|....|....|....|....|....|....|....|....|....|....|....|....|
Trinbago virus ATCGTCACGCCCACTGCCAGTGGAAAAAGCACTCTCTTCCCGCTCGAGCTGGCAATGGAGATGGTGGAGCGAAAAAACTTTGGCGCAATCATTGTGCTCA
p3             ----------------------------------------------------------------------------------------------------
p11            ----------------------------------------------------------------------------------------------------
p43            ----------------------------------------------------------------------------------------------------
p53            ----------------------------------------------------------------------------------------------------
p57            ----------------------------------------------------------------------------------------------------
p70            ----------------------------------------------------------------------------------------------------
p81            ----------------------------------------------------------------------------------------------------
p83            ATCGTCACGCCTACTGCCAGTGGAAAGAGCACCCTCTTCCCGCTCGAGCTGGCAATGGAGATGGTTGAGCGGAAGAACTTTGTCTCAATCATTGTGCTCA

                       5810      5820      5830      5840      5850      5860      5870      5880      5890      5900
               ....|....|....|....|....|....|....|....|....|....|....|....|....|....|....|....|....|....|....|....|
Trinbago virus ACCCTCTCCGAGCGACCACCAGTGGGCTGGCAGCCTACGTGCGAGGACTTGTCACGGCCCGAGGCCTGGCAAGCAAGATTACTGTTGAATCCGCCATCGG
p3             ----------------------------------------------------------------------------------------------------
p11            ----------------------------------------------------------------------------------------------------
p43            ----------------------------------------------------------------------------------------------------
p53            ----------------------------------------------------------------------------------------------------
p57            ----------------------------------------------------------------------------------------------------
p70            ----------------------------------------------------------------------------------------------------
p81            ----------------------------------------------------------------------------------------------------
p83            ACCCTCTCCGAGCGACCAC---------------------------------------------------------------------------------
                       6410      6420      6430      6440      6450      6460      6470      6480      6490      6500
               ....|....|....|....|....|....|....|....|....|....|....|....|....|....|....|....|....|....|....|....|
Trinbago virus GCAGCAGGAAGGCCATAATCGTTGCCACCAATGCCATTGAGAGTGGCATTACCCTGAACGACCTGACCGATGTCTGGGACACTCAGGAGGAGAACGTTGT
p3             ----------------------------------------------------------------------------------------------------
p11            ----------------------------------------------------------------------------------------------------
p43            ----------------------------------------------------------------------------------------------------
p53            ----------------------------------------------------------------------------------------------------
p57            ----------------------------------------------------------------------------------------------------
p70            -------------CATCATTGTTGGCACCAATGCCCTTGAGAGTGGCATTACCCTGAATGACCTGACCGATGTCTGGGACACTCAGGAGGAGAACGTTGT
p81            ----------------------------------------------------------------------------------------------------
p83            ----------------------------------------------------------------------------------------------------
```

```
                      6510      6520      6530      6540      6550      6560      6570      6580      6590      6600
              ....|....|....|....|....|....|....|....|....|....|....|....|....|....|....|....|....|....|....|....|
Trinbago virus CGAAGTCGAAGACAACGGCAACACCTGCTTCAAAAGGAGGCCAATCTCCAAAAAGAGCGCCATCCAACGGCGCGGTCGCTGCGGTAGGACCACCGACACC
p3             ----------------------------------------------------------------------------------------------------
p11            ----------------------------------------------------------------------------------------------------
p43            ----------------------------------------------------------------------------------------------------
p53            ----------------------------------------------------------------------------------------------------
p57            ----------------------------------------------------------------------------------------------------
p70            CGAAGTCGAAGACAACGGCAACACCTGCTTCAAGAGGAGGCCAATCTCCAAGAAGAGCGCCATCCACCGGCGCGGCCGCTGCGGTAGGACCACCGACACC
p81            ----------------------------------------------------------------------------------------------------
p83            ----------------------------------------------------------------------------------------------------
                      6610      6620      6630      6640      6650      6660      6670      6680      6690      6700
              ....|....|....|....|....|....|....|....|....|....|....|....|....|....|....|....|....|....|....|....|
Trinbago virus GCAAACTACTGGTTCCCACGGGGCAATGTGCTTCCCACGGACCAGCCAATCGACTATGAGATCAAGCTGCTTTGTGCAGCGCATTGGTTGGCCGTCTCCA
p3             ----------------------------------------------------------------------------------------------------
p11            ----------------------------------------------------------------------------------------------------
p43            ----------------------------------------------------------------------------------------------------
p53            ----------------------------------------------------------------------------------------------------
p57            ----------------------------------------------------------------------------------------------------
p70            GCAAACTACTGGTTCCCACGGGGTAACGTGCTTCCCATGGACCAGCCAATCGACTACCA-----------------------------------------
p81            ----------------------------------------------------------------------------------------------------
p83            ----------------------------------------------------------------------------------------------------
                     10410     10420     10430     10440     10450     10460     10470     10480     10490     10500
              ....|....|....|....|....|....|....|....|....|....|....|....|....|....|....|....|....|....|....|....|
Trinbago virus GTGTTCATCCGTCTACATCGTGCATGAGCGCCTTGCTGCCATGCGGGGAAACCTGGGCAACACGACCTACGACGACTCACCCCTCCCCCTCCCGGTGTCT
p3             ----------------------------------------------------------------------------------------------------
p11            ----------------------------------------------------------------------------------------------------
p43            ---------------------------------------------------------------------------------------CCTCCCGGTGTCT
p53            ----------------------------------------------------------------------------------------------------
p57            ----------------------------------------------------------------------------------------------------
p70            ----------------------------------------------------------------------------------------------------
p81            ----------------------------------------------------------------------------------------------------
p83            ----------------------------------------------------------------------------------------------------

                     10510     10520     10530     10540     10550     10560     10570     10580     10590     10600
              ....|....|....|....|....|....|....|....|....|....|....|....|....|....|....|....|....|....|....|....|
Trinbago virus GTCGGCAGCTGGGACAAGAGTGAGATCGACACATACCAGCAGGTGGAATCGTCTAACGCCATCGCTAGGTACGTCGCTGACATCACCAGGGACGCAGACA
p3             ----------------------------------------------------------------------------------------------------
p11            ----------------------------------------------------------------------------------------------------
p43            GTTTGCAGTTGGGACAAGAGCGAGATTGACACTTACCAGCAGGTGGAATCGTCCAACGCCATCGCGAGGTACGTCGCTGAAATCACCAAAAACAAGGACC
p53            ----------------------------------------------------------------------------------------------------
p57            ----------------------------------------------------------------------------------------------------
p70            ----------------------------------------------------------------------------------------------------
p81            ----------------------------------------------------------------------------------------------------
p83            ----------------------------------------------------------------------------------------------------

                     10610     10620     10630     10640     10650     10660     10670     10680     10690     10700
              ....|....|....|....|....|....|....|....|....|....|....|....|....|....|....|....|....|....|....|....|
Trinbago virus TCATTGTCGAGCCTTGTGCGGGGCTGGGTCGTCTCACAGCGCTCTGCGCATACCAAAACCATACCTCGCTCTGCCTCTCTGACATCTCAGTGCACTGCAC
p3             ----------------------------------------------------------------------------------------------------
p11            ----------------------------------------------------------------------------------------------------
p43            TCATCATCGAGCCTTGTGCGGGGCTGGGCCGTCTCACAGCTCTCTGCGCGTACCAAAATCGAACCCCGCTCTGCCTCTCTGACATTTCATTGCACTGCGT
p53            ----------------------------------------------------------------------------------------------------
p57            ----------------------------------------------------------------------------------------------------
p70            ----------------------------------------------------------------------------------------------------
p81            ----------------------------------------------------------------------------------------------------
p83            ----------------------------------------------------------------------------------------------------
```

```
                    10710     10720     10730     10740     10750     10760     10770     10780     10790     10800
               ....|....|....|....|....|....|....|....|....|....|....|....|....|....|....|....|....|....|....|....|
Trinbago virus TGAGCAGCTGCGGCACCTTTACGAACGCCCCGAGTGCCCGGCCAAAGTGGTCATCGAGCACAAGGATGTCATGGCCTTGGGCAAACAACTCAAAGGGCAG
p3             ----------------------------------------------------------------------------------------------------
p11            ----------------------------------------------------------------------------------------------------
p43            TGAGCAGCTGCGGCACCTGTACGAACGCCCCGAGTGCCCGGTCAAAGTGGTTATCGAGCACAGGGTTGTCATGAGTCTGGGCAAACAGCTCAAAGGGCAG
p53            ----------------------------------------------------------------------------------------------------
p57            ----------------------------------------------------------------------------------------------------
p70            ----------------------------------------------------------------------------------------------------
p81            ----------------------------------------------------------------------------------------------------
p83            ----------------------------------------------------------------------------------------------------

                    10810     10820     10830     10840     10850     10860     10870     10880     10890     10900
               ....|....|....|....|....|....|....|....|....|....|....|....|....|....|....|....|....|....|....|....|
Trinbago virus ACGGTGGTGTGCAACCCACCATATAACCGGCCAGAAGATACCACTCTTGGAACGAGGGTGTGTAAAGCCATCTTCAAGCAGAGCCCATCACGGTTCATTT
p3             ----------------------------------------------------------------------------------------------------
p11            ----------------------------------------------------------------------------------------------------
p43            ACGGTGGTTTGCAACCCACAC-------------------------------------------------------------------------------
p53            ----------------------------------------------------------------------------------------------------
p57            ----------------------------------------------------------------------------------------------------
p70            ----------------------------------------------------------------------------------------------------
p81            ----------------------------------------------------------------------------------------------------
p83            ----------------------------------------------------------------------------------------------------
                    12010     12020     12030     12040     12050     12060     12070     12080     12090     12100
               ....|....|....|....|....|....|....|....|....|....|....|....|....|....|....|....|....|....|....|....|
Trinbago virus GAGTGGACGCTGATCAAGAGTGAGGAGGCCGAACACCAGGACTTCTCACGCCAGGCAAACCGCGCGAAAGTGCGACAACCAGTCGAACTCAAGGGTGGCT
p3             ----------------------------------------------------------------------------------------------------
p11            -----------------------------------------CTTCTCACGCCTGTCCAATCGCGCGAAAGTGCGGCAACCAGTTGAACTCAAGGGTGGCT
p43            ----------------------------------------------------------------------------------------------------
p53            ----------------------------------------------------------------------------------------------------
p57            ----------------------------------------------------------------------------------------------------
p70            ----------------------------------------------------------------------------------------------------
p81            ----------------------------------------------------------------------------------------------------
p83            ----------------------------------------------------------------------------------------------------
                    12110     12120     12130     12140     12150     12160     12170     12180     12190     12200
               ....|....|....|....|....|....|....|....|....|....|....|....|....|....|....|....|....|....|....|....|
Trinbago virus GGCCAGGCTATGAGGGACTCAAGGGCCAATTCAAGAACAGGCTCGACAGGCTCGAGGAAACATGCGCTCACTACAAGATCAAGCTGCAACCCGCAAGCAG
p3             ----------------------------------------------------------------------------------------------------
p11            GGCCAGGTTATGAAGGACTCAAAGCCCAATTCAAGAACAGACTCGACAGACTCGCGGAAACATGCACCCACTACAAGATCAAGTTGCAACCCGCAAGCAG
p43            ----------------------------------------------------------------------------------------------------
p53            ----------------------------------------------------------------------------------------------------
p57            ----------------------------------------------------------------------------------------------------
p70            ----------------------------------------------------------------------------------------------------
p81            ----------------------------------------------------------------------------------------------------
p83            ----------------------------------------------------------------------------------------------------

                    12210     12220     12230     12240     12250     12260     12270     12280     12290     12300
               ....|....|....|....|....|....|....|....|....|....|....|....|....|....|....|....|....|....|....|....|
Trinbago virus AGCGCGTGTCTTCCAAAATCAAGAAGTCTTGGGCACGTACAACAAAAATGTCCGATTACAGGAGGACAAGACAATGCTGCAACAAACCACCTATCGTGCA
p3             ----------------------------------------------------------------------------------------------------
p11            AGCGCGTGTATTCCAAAACCAAGAAGTCTTGGGCACGTTCAACAAGAATGTCCGACTACAAGAGGACAAAACAATGCTGCAACAGACCACCTATCGTGCA
p43            ----------------------------------------------------------------------------------------------------
p53            ----------------------------------------------------------------------------------------------------
p57            ----------------------------------------------------------------------------------------------------
p70            ----------------------------------------------------------------------------------------------------
p81            ----------------------------------------------------------------------------------------------------
p83            ----------------------------------------------------------------------------------------------------
```

```
                    12310     12320     12330     12340     12350     12360     12370     12380     12390     12400
               ....|....|....|....|....|....|....|....|....|....|....|....|....|....|....|....|....|....|....|....|
Trinbago virus GCAAACACCATCTGGGAGTGGGACCCCCGCTGCTCAAACGTGAAACAAGTGAGCAGAGACCGCGTCGAGGTCCTTGGGGCGATTCAAGATCGTATCGACG
p3             ----------------------------------------------------------------------------------------------------
p11            GCGAATGCTATCTGGGAGTGGGACCCCCGCTGCTCGAACGTCAAACAAGTGAGCAGAGACCGCGTTGAGGTCCTTGGGGCAATCCAAGATCGCATTGACG
p43            ----------------------------------------------------------------------------------------------------
p53            ----------------------------------------------------------------------------------------------------
p57            ----------------------------------------------------------------------------------------------------
p70            ----------------------------------------------------------------------------------------------------
p81            ----------------------------------------------------------------------------------------------------
p83            ----------------------------------------------------------------------------------------------------
                    12410     12420     12430     12440     12450     12460     12470     12480     12490     12500
               ....|....|....|....|....|....|....|....|....|....|....|....|....|....|....|....|....|....|....|....|
Trinbago virus CCCCACACTACTTCCCAGCCCCTGATGTCCTGGAAGAACTGCACACCATCGCAGGCCACATCCGGACAAAAACGAACATCAAACTCAGGCCATACACATT
p3             ----------------------------------------------------------------------------------------------------
p11            CCCCACACTACTTCCCAGCCCCTGATGTCCTGGAAGAACTGCACACCATCGCAGGCCACATCCGGACAAAAACGAAC-----------------------
p43            ----------------------------------------------------------------------------------------------------
p53            ----------------------------------------------------------------------------------------------------
p57            ----------------------------------------------------------------------------------------------------
p70            ----------------------------------------------------------------------------------------------------
p81            ----------------------------------------------------------------------------------------------------
p83            ----------------------------------------------------------------------------------------------------
                    12610     12620     12630     12640     12650     12660     12670     12680     12690     12700
               ....|....|....|....|....|....|....|....|....|....|....|....|....|....|....|....|....|....|....|....|
Trinbago virus ATCTGCGAGGAGGCCATCCAAAAACTGTACCGGGGAGAAAATGTTGTGGAATATTACAACACAGTCCACAACAAAGTTGAATCCAAGGTGAGCGATAACA
p3             ----------------------------------------------------------------------------------------------------
p11            ----------------------------------------------------------------------------------------------------
p43            ----------------------------------------------------------------------------------------------------
p53            -------------------------------------------------------------------------------AGAGTAAGGTGACCAAGAACA
p57            ----------------------------------------------------------------------------------------------------
p70            ----------------------------------------------------------------------------------------------------
p81            ----------------------------------------------------------------------------------------------------
p83            ----------------------------------------------------------------------------------------------------

                    12710     12720     12730     12740     12750     12760     12770     12780     12790     12800
               ....|....|....|....|....|....|....|....|....|....|....|....|....|....|....|....|....|....|....|....|
Trinbago virus TCCGCCCCCGTCTTATCAACTACGGCAGCATCGTGGCCCGCGTCTGCGACCACATGCTATTCGGACCCTTCGTGAAGCTCCACTACGGTGGAACCAAGCT
p3             ----------------------------------------------------------------------------------------------------
p11            ----------------------------------------------------------------------------------------------------
p43            ----------------------------------------------------------------------------------------------------
p53            CCCGGGCGCGGCGCATCAACTATGGCAGCATCGTGGCCCGCGTGGTTGACCATATGCCCTTCGGTCCTTTCGTSAAGCTGCACTATTCTGGCCAGAAGCT
p57            ----------------------------------------------------------------------------------------------------
p70            ----------------------------------------------------------------------------------------------------
p81            ----------------------------------------------------------------------------------------------------
p83            ----------------------------------------------------------------------------------------------------

                    12810     12820     12830     12840     12850     12860     12870     12880     12890     12900
               ....|....|....|....|....|....|....|....|....|....|....|....|....|....|....|....|....|....|....|....|
Trinbago virus TTTCAAACACGCAAGCGGAGGAACCCCGATCGCCGAGATGGGAGACCGCATCAAGAAGCATTGGGACCACTGGGCTTCCAAGTGCGTGGGGGATGAAGAT
p3             ----------------------ACCCCAATCGCGGAGATGGGTGACCGCCTTAAGAAGCACTGGGATCACTGGGCTTCCAGGTGCGTTGGAGATGAAGAA
p11            ----------------------------------------------------------------------------------------------------
p43            ----------------------------------------------------------------------------------------------------
p53            ATTCAAGCACGYCAGTGGTGGCACCCCCATCACAGAAATGGGTGTTCGCATCAAGAAACACTGGGTCAAGTTGGTGGCCAAGTGCACAGTCGAGGA----
p57            ----------------------------------------------------------------------------------------------------
p70            ----------------------------------------------------------------------------------------------------
p81            ----------------------------------------------------------------------------------------------------
p83            ----------------------------------------------------------------------------------------------------
```

```
                     12910     12920     12930     12940     12950     12960     12970     12980     12990     13000
             ....|....|....|....|....|....|....|....|....|....|....|....|....|....|....|....|....|....|....|....|
Trinbago virus CCTGTGGCCGCAGTCGGCGACGCATCACGCTGGGACCACAGTATGTACCCAGCTCTCATGGCACTGGAGGCTGAGTTTGTTTCAAGCTTCTATGACAAAG
p3             CCTGTGGCCGCAGTCGGCGACGCATCACGCTGGGATCACAGCATGTACCCAGCTCTCATGGCATTGGAGGCTGAGTTTGTCTCAAGCTTCTATGACAAAG
p11            ----------------------------------------------------------------------------------------------------
p43            ----------------------------------------------------------------------------------------------------
p53            ----------------------------------------------------------------------------------------------------
p57            ----------------------------------------------------------------------------------------------------
p70            ----------------------------------------------------------------------------------------------------
p81            ----------------------------------------------------------------------------------------------------
p83            ----------------------------------------------------------------------------------------------------
                     13010     13020     13030     13040     13050     13060     13070     13080     13090     13100
             ....|....|....|....|....|....|....|....|....|....|....|....|....|....|....|....|....|....|....|....|
Trinbago virus AGCACTGGCCGGCCATACAGGCAGCACTCGAACACGTCTGCTGGCCTTTGTGTTTCACAAGGCTTGGCTTCGTTTTCACCAACCCAGGCCAGAGGATGAG
p3             AGCACTGGCCGGCCATACAAGCAGCACTCGAACATGTCTGCTGGCCTTTGTGTTTCACAAGGCTTGGCTTCGTTTTCACCAACCCAGGCCAGAGGATGAG
p11            ----------------------------------------------------------------------------------------------------
p43            ----------------------------------------------------------------------------------------------------
p53            ----------------------------------------------------------------------------------------------------
p57            ----------------------------------------------------------------------------------------------------
p70            ----------------------------------------------------------------------------------------------------
p81            ----------------------------------------------------------------------------------------------------
p83            ----------------------------------------------------------------------------------------------------

                     13110     13120     13130     13140     13150     13160     13170     13180     13190     13200
             ....|....|....|....|....|....|....|....|....|....|....|....|....|....|....|....|....|....|....|....|
Trinbago virus CGGGCACGTGTTCACTTGGATGAACTCGTTCCTCAACGCAACGCTGCACGAGTGGGGGTGGAAAAAGACCCTCAATATCCCCCTCGAAGCAGACCTTGAC
p3             CGGGCACGTGTTCACCTGGATGAACTCATTCCTCAATGCCACGCTGCACGAGTGGGGGTG----------------------------------------
p11            ----------------------------------------------------------------------------------------------------
p43            ----------------------------------------------------------------------------------------------------
p53            ----------------------------------------------------------------------------------------------------
p57            ----------------------------------------------------------------------------------------------------
p70            ----------------------------------------------------------------------------------------------------
p81            ----------------------------------------------------------------------------------------------------
p83            ----------------------------------------------------------------------------------------------------
                     13810     13820     13830     13840     13850     13860     13870     13880     13890     13900
             ....|....|....|....|....|....|....|....|....|....|....|....|....|....|....|....|....|....|....|....|
Trinbago virus GCGTATGAAGAACGCGCAGAAGACATCAGAAGTAGAGAGGCAGCCGTAACCACCGCCGATGAATTATTTGAGGCTGACTTCCACCTTGAGGCCCAAGCAG
p3             ----------------------------------------------------------------------------------------------------
p11            ----------------------------------------------------------------------------------------------------
p43            ----------------------------------------------------------------------------------------------------
p53            ----------------------------------------------------------------------------------------------------
p57            --------------------------------------GGCAGCCGTCATCACTGCCGACGAATTACTTGAGACTGACTTTCACCTTGAGGCCCAGGCAG
p70            ----------------------------------------------------------------------------------------------------
p81            ----------------------------------------------------------------------------------------------------
p83            ----------------------------------------------------------------------------------------------------

                     13910     13920     13930     13940     13950     13960     13970     13980     13990     14000
             ....|....|....|....|....|....|....|....|....|....|....|....|....|....|....|....|....|....|....|....|
Trinbago virus CGACCGGTATTGTCGTCGGAGCACTCCGATCAATCTACGGGCAATCCATCCAAAGCCTGGACGACATCGGATGCGTCGACTTCCGCTGGGAGAGAACAAG
p3             ----------------------------------------------------------------------------------------------------
p11            ----------------------------------------------------------------------------------------------------
p43            ----------------------------------------------------------------------------------------------------
p53            ----------------------------------------------------------------------------------------------------
p57            CAACCGGCATCGTTGTCGGAGCGCTCCGATCAATCTACGGGCAATCCATCCAAAGCCTGGACGACATCGGATGCGTCGACTTCCGCTGGGAGAGAGCAAG
p70            ----------------------------------------------------------------------------------------------------
p81            ----------------------------------------------------------------------------------------------------
p83            ----------------------------------------------------------------------------------------------------
```

```
                        14010     14020     14030     14040     14050     14060     14070     14080     14090     14100
                ....|....|....|....|....|....|....|....|....|....|....|....|....|....|....|....|....|....|....|....|
Trinbago virus  CTGCCGCGACATGATCACCCTCATGGTCAATGGCGCGAAGGCCGGCGCAGGCAACTCCATAACGCCTGACATGGAACGATGCGCTGTGGACGCAAAGTTC
p3              ----------------------------------------------------------------------------------------------------
p11             ----------------------------------------------------------------------------------------------------
p43             ----------------------------------------------------------------------------------------------------
p53             ----------------------------------------------------------------------------------------------------
p57             CTGCCGCGACATGATCACCCTCATGGTCAATGGCGCGAAGGCCGGTGCAGGCAACTCCATCACACCTG--------------------------------
p70             ----------------------------------------------------------------------------------------------------
p81             ----------------------------------------------------------------------------------------------------
p83             ----------------------------------------------------------------------------------------------------
```
