# Supplementary material for: Metagenomic Investigation of Ticks From Kenyan Wildlife Reveals Diverse Microbial Pathogens and New Country Pathogen Records
Source: Front Microbiol. 2022 Jul 1;13:932224. doi: 10.3389/fmicb.2022.932224 (PMC9283121; doi:10.3389/fmicb.2022.932224)
Supplement: Supplementary file 3 [file Data_Sheet_2.PDF]

Supplement 3: Alignment of the Guarapuava tymovirus-like 1 virus sequences detected in pools 52, 53 and 83. Positions are given according to the isolate Kenya-P43 (OM807119).

```
              3310      3320      3330      3340      3350      3360      3370      3380      3390      3400
      ....|....|....|....|....|....|....|....|....|....|....|....|....|....|....|....|....|....|....|....|
p43   CTTGACAGCTCCACCTTTTCTGCCACCCTCACAGGCGGCTCCAAAGTCGTGCGCTCCTCCAGCSAAATGTTTGGCCCCCCTGGGCTCGACCCCGAGTATT
p53   ---------------------------------------------------------------------------CCGGTTTCATTTTTGCAGGAGTACT
p52   ----------------------------------------------------------------------------------------------------
p83   ----------------------------------------------------------------------------------------------------

              3410      3420      3430      3440      3450      3460      3470      3480      3490      3500
      ....|....|....|....|....|....|....|....|....|....|....|....|....|....|....|....|....|....|....|....|
p43   CCGCCGATGTCCTTCGAGGGGAGCGCGTTCAAGAAGGCTCTTCAGAACGTGCCACCACGCTGCACCTCCCCCCCAGTCGTCTCCCGCTCCATCAGGACCT
p53   CCGCCGACGTGCTTCGCGGGGAACTCGTTCAGGAAGGCTCTCCCGAACGCGCCACCACGCTTCACCTTCCACCCAGCCGCCTCCCTCTTCACCAGGACCT
p52   ----------------------------------------------------------------------------------------------------
p83   ----------------------------------------------------------------------------------------------------

              3510      3520      3530      3540      3550      3560      3570      3580      3590      3600
      ....|....|....|....|....|....|....|....|....|....|....|....|....|....|....|....|....|....|....|....|
p43   CGAAATCCTTACGCCCAGCTCAGCGGACCCACTTGGTACGTCCTTCACCCCTCCTTTCGCGGAACCTTGCATTCCCAGAGTTGACTTTAGGGACCTCGTC
p53   TGAAATCCTCACACCCAGTCCCGCAAACCCCCCCGACGCCCTCCCCTGGCCCCCATTCGCAGAGCCCCCCATCCCCAGAGTCGACTTCAGAGACAAGGTC
p52   ----------------------------------------------------------------------------------------------------
p83   ----------------------------------------------------------------------------------------------------

              3610      3620      3630      3640      3650      3660      3670      3680      3690      3700
      ....|....|....|....|....|....|....|....|....|....|....|....|....|....|....|....|....|....|....|....|
p43   ACTACCTTCGACTCCGAGCCATGGGACGCCTCCGCTAAAGAAATCACCTTCCGAGGAGTGCTTTCCAACCAGTTCCCCGAGCTTAACGCTCCCAGGCTTT
p53   ACCTCCTTCGACTCCGAACCCTGGGATGCGTCGGCCAAGGAGATCGTCTTCCGGGGAGTTCTCTCTAACCAGTTCCCGGAGCTCAACGCTCCCAGGCTTC
p52   ----------------------------------------------------------------------------------------------------
p83   ----------------------------------------------------------------------------------------------------

              3710      3720      3730      3740      3750      3760      3770      3780      3790      3800
      ....|....|....|....|....|....|....|....|....|....|....|....|....|....|....|....|....|....|....|....|
p43   TGGGAGCGCAGGCCCTCGGCACCATCGCAGCCCGCCACGACGAACGGAGGGACCCCACACTCCTCAAGGCCAGCATAACAAAGAGGTTGCGCTTTAGACG
p53   TGGGAGCGCAGGCCCTCGGCACCATTGCCGCCCGCCACGACGAACGGAGGGACCCAACTCTCCTCAAAGCCAGCATAACCAAGAGGTTGCGCTTTCGACG
p52   TGGGAGCGCAGGCCCTCGGCACCATCGCAGCCCGCCACGACGAACGGAGGGACCCCACACTCCTCAAGGCCAGCATAACAACGAGGTTGCGCTTTAGACG
p83   ----------------------------------------------------------------------------------------------------

              3810      3820      3830      3840      3850      3860      3870      3880      3890      3900
      ....|....|....|....|....|....|....|....|....|....|....|....|....|....|....|....|....|....|....|....|
p43   CGACAGCCGACCTTACCGGTTCTCCGACCGAGACCACACACTCGCTCACCTGCTCATCTCCTCGCTCGCCAGTTTGTACCACCGCGACCCCAATCGCCGC
p53   CAACAGTCAGCCCTACCGTTTCTCCGACCGCGACCACACCCTCGCCCACTTGCTCATCACTTCTCTAGCCGAGCTGTACGGCCGTGACCCCAACCGCCGC
p52   CGACAGCCGACCTTACCGGTTCTCCGACGGTGTCCTCACACTCGCTCACCTGCTCATCTCCTCGCTCGCCGGTTTGTACCACCGCGACCCCAATCGCCGC
p83   ----------------------------------------------------------------------------------------------------
```

```
               3910      3920      3930      3940      3950      3960      3970      3980      3990      4000
      ....|....|....|....|....|....|....|....|....|....|....|....|....|....|....|....|....|....|....|....|
p43   GAACCCTTTGACCCCGACCTGTTCGCCCGCTGCATCGCCGAGAACGAATTCGCCCAGCTCACTAACAAAACCCAAAAGGTGATCATGGCCAACGCTGAGC
p53   GAACCCTTCGACCCCGACTTATTCGCCCGTTGCATCGCGGAAAACGAGTTCGCCCAGCTCACCAACAAAACTCAGAAGGTGATCATGGCCAACGCGGAGC
p52   GAACCCTTTGACCCCGATCTTTTCGCCCGTTGCATCGCGGAAAATGAATTCGCTCATCTCACCAACAAAACCCAGAAGGTGATCATGGACAACGCGGTGC
p83   ----------------------------------------------------------------------------------------------------

               4010      4020      4030      4040      4050      4060      4070      4080      4090      4100
      ....|....|....|....|....|....|....|....|....|....|....|....|....|....|....|....|....|....|....|....|
p43   GGTCGGATCCAGACTGGCGGTGGAGTGCCGTCCGCATCTTCGCTAAGACGCAACATAAAATCAACGCCGGCACCATCTTCGGCCCTTGGAAAGCCTGTCA
p53   GCTCAGACCCGGATTGGCGGTGGAGTGTGGTCCGCATCTTCGCCAAGACGCAGCACAAAATCAATGCCGGCACCATCTTCGGCCCCTGGAAGGCCTGCCA
p52   GTTCAGTCCCTGACTGGCGTTGGAGTGTCGTCCGCA----------------------------------------------------------------
p83   ----------------------------------------------------------------------------------------------------

               4110      4120      4130      4140      4150      4160      4170      4180      4190      4200
      ....|....|....|....|....|....|....|....|....|....|....|....|....|....|....|....|....|....|....|....|
p43   GACTCTCGCTTTGATGCATGACGCTGTCATTCTTCTGCTTGGCCCAGTCAAGAAGTATCAGCGCCTCTTTGACGCCAAAGATCGCCCGCCCAACATCTAC
p53   AACCCTCGCCCTGATGCACGACGCGGTCATCCTTCTTCTCGGGCCCGTTAAGAAATACCAAAGCCTCTTTGATGGCAAAGATCGCCCACCCAACATTTAC
p52   ----------------------------------------------------------------------------------------------------
p83   ----------------------------------------------------------------------------------------------------

               4210      4220      4230      4240      4250      4260      4270      4280      4290      4300
      ....|....|....|....|....|....|....|....|....|....|....|....|....|....|....|....|....|....|....|....|
p43   ATCCACGCCGGGAAGACCCCGTCTCAGCTCAGCGCCTACTGCCAGAGCCGGCTCCGCTCTTCCAGTTCGATCGCAAACGACTACACCGCGTTCGACCAAT
p53   ATCCACGCCGGAAAAACTCCCTCCCAGCTTAGCTCCTTCTGCCAGAGCCGGCTTCGTTCCTCTACCTCGATCGCCAACGACTACACAGCATTCGATCAAT
p52   ----------------------------------------------------------------------------------------------------
p83   ----------------------------------------------------------------------------------------------------

               4310      4320      4330      4340      4350      4360      4370      4380      4390      4400
      ....|....|....|....|....|....|....|....|....|....|....|....|....|....|....|....|....|....|....|....|
p43   CCCAGCATGGGGAGGCGGTCTTACTCGAGCGCTGGAAGATGTGGCGCCTTTCCATCCCCGATCATCTCATCCGCCTCCACGTCTGGATCAAAACGAACAT
p53   CCCAGCATGGCGAAGCCGTCCTGCTCGAGCGCTGGAAAATGTGGCGCCTTTCCATCCCTGAGGAGCTCATCCGCCTGCATGTCTGGATCAAAACTAACAT
p52   ----------------------------------------------------------------------------------------------------
p83   ----------------------------------------------------------------------------------------------------

               4410      4420      4430      4440      4450      4460      4470      4480      4490      4500
      ....|....|....|....|....|....|....|....|....|....|....|....|....|....|....|....|....|....|....|....|
p43   CACGACGCAGTTCGGTCCCCTCACTTGCATGCGCCTCACTGGAGAACCCGGCACCTACGATGACAACAGCGACTACAACCTCGCTGTTCTGGGCCTTCGT
p53   CTCCACTCAGTTTGGTCCTCTCACGTGCATGCGTCTCACCGGCGAACCAGGCACCTACGACGACAACAGCGACTACAATCTAGCTGTTCTCGGCCTTCGT
p52   ----------------------------------------------------------------------------------------------------
p83   -------------------------------------------------------------------------------------GTGTTGGGACGAAAA

               4510      4520      4530      4540      4550      4560      4570      4580      4590      4600
      ....|....|....|....|....|....|....|....|....|....|....|....|....|....|....|....|....|....|....|....|
p43   TACCAGCTTTCGCCCCACCACACCGTCTTCGTGAGCGGCGACGACTCCGCCGTTTTCCCGCCTCCTCGCGAGCACCCCCATTGGGCTCACACCAAGCCCC
p53   TACCTGCTCTCTCCCCAACACACCATCTTCGTGAGTGGCGATGACTCCGCTGTCTTCCCACCTCCTCGCGAGCATCCTCGTTGGGCCCACACGAAACCTC
p52   ----------------------------------------------------------------------------------------------------
p83   GAGGTAGCGAAGGCCCAGGACAGCATTTTCGTGAGTGGAGATGACTCCGCCATCTTTCCGCCCCCTCGCGAACATCCTCGTTGGGCTCACACTAAACCTC
```

```
               4610      4620      4630      4640      4650      4660      4670      4680      4690      4700
        ....|....|....|....|....|....|....|....|....|....|....|....|....|....|....|....|....|....|....|....|
p43     TTCTGCATCTTCGCTTCAAGACCGTCCAACAGCAGCACACGCTTTTCTGCGGCTACTACCTGGGACCTGCTGGCGCCTGCCGAGACCCTTTAGCCCTGTT
p53     TTCTTCACCTCCGTTTCAAGACTGTCCAGCAGACGCACACCCTCTTCTGCGGCTATTACCTTGGTCCAGCGGGCGCCTGCCGAGATCCCCGGGCTCTGTG
p52     ----------------------------------------------------------------------------------------------------
p83     TTCTCCACCTCCGCTTCAAGACCGTCCAGCAGGAACACACGCTCTTCTGCGGTTACTACCTCGGTCCGGCCGGCGCCTGCCGAGATCCCCTCGCCCTCTT

               4710      4720      4730      4740      4750      4760      4770      4780      4790      4800
        ....|....|....|....|....|....|....|....|....|....|....|....|....|....|....|....|....|....|....|....|
p43     CGCCAAGCTCGCGGTAGCCCAGGATGAGGACCGGCTCCAGGAAGTGCTGCTCTCTCACTTGGCAGAATTTAGCACCGGCCATCGCCTTGGGGAGCCGGTC
p53     CGCCAAGCTCGCCGTCGCGCAGGACGAGGACCGGCTCCAGGACGTCCTGCTTTCACACCTCGCCGAATTCAGCACCGGCCATCGCCTCGGCGAGCCGGTC
p52     ----------------------------------------------------------------------------------------------------
p83     CGCCAAACTCGCCGTCGCACAGGACGAAGATCGGCTTCAGGAAGTGCTACTTTCTCACCTCGCCGAATTTAGCACCGGCCATCGTCTCGGTGAACCAGTC

               4810      4820      4830      4840      4850      4860      4870      4880      4890      4900
        ....|....|....|....|....|....|....|....|....|....|....|....|....|....|....|....|....|....|....|....|
p43     TTTTCACTCTTCCCGGAGTCATTGACCCTTTACCACTGCGCTTGCTTTCAGCTCTTTTGCCAAAAATGCTCCCCAGCGCAAAAGCTCATTTTGCGTGCCC
p53     TTTTCGCTCTTCCCTGAATCGCTCAGTCTCTACCACTGCGCCTGCTTCCAGCTCTTCTGCCAAAAATGCTCCCCAGCTCAAAAGTTAATCCTGCGTGCGC
p52     ----------------------------------------------------------------------------------------------------
p83     TTCTCACTCTTTCCCGAATCGCTCAGCCTCTACCACTGCGCTTGCTTCCAGCTCTTCTGTCAGAAGTGCTCCCCCGCCCAGAAGCTGATTCTGCGTGCGC

               4910      4920      4930      4940      4950      4960      4970      4980      4990      5000
        ....|....|....|....|....|....|....|....|....|....|....|....|....|....|....|....|....|....|....|....|
p43     CCGGCACCGCCATCGGCAGCATTTCAAAATCCATCACCGCTTCCGATCGACTCTCACAGAAGGCCTATCAGATCCTTCGGGAATTGGACCCCAACTTTCA
p53     CTGGCACTGCCATTGGCAGCATTTCCAAAACCATCACAGCCTCCGATCGACTTTCACAAAAAGCTTACCAGATCCTCCGGGAATTGGACCCTAACTTCCA
p52     ----------------------------------------------------------------------------------------------------
p83     CCGGCACCGCAATTGACAGCATCTCCAAAACAATCACAGCCTCTGACCGACTTTCACAGAAAGCCTACCAG-----------------------------

               5010      5020      5030      5040      5050      5060      5070      5080      5090      5100
        ....|....|....|....|....|....|....|....|....|....|....|....|....|....|....|....|....|....|....|....|
p43     GCACCATAATTTCTCGGATGGCCCATATTAGTTGTTGCCAATGAGTTTCGCCGTCATGGCCCTACCCGCTCTCTTCGGTTTAGTGGAAACCATAGTCGAC
p53     GCATCACAACTTTTCCGACCGACCCTATTAGTTGTTGCCACTTGGTTTCGCCGTCATGGCCTTGCCCGCGATCTTTGGTTTGGTAGAAACCCTCGTAGAG
p52     ----------------------------------------------------------------------------------------------------
p83     ----------------------------------------------------------------------------------------------------

               5110      5120      5130      5140      5150      5160      5170      5180      5190      5200
        ....|....|....|....|....|....|....|....|....|....|....|....|....|....|....|....|....|....|....|....|
p43     TCTGCAGAGGTGCTCTCTCTTGCTGAACCCGCGGTATCACTAGTCTCTTCCTTAGCTTCTTCTAACCCTTCGCCTTCGGCCGCCGAGTCGGAAGAGCCCT
p53     TCATCGGAAGTGCTCTCTTTAGTCGAGCCAGCTATTTCACTCGCTTCCTCATTCTCCTCTTCGAACCCATCCCCCTC-----------------------
p52     ----------------------------------------------------------------------------------------------------
p83     ----------------------------------------------------------------------------------------------------
```
